# Targeting the PREX2/RAC1/PI3Kβ Signaling Axis Confers Sensitivity to Clinically Relevant Therapeutic Approaches in Melanoma

**DOI:** 10.1158/0008-5472.CAN-23-2814

**Published:** 2024-12-05

**Authors:** Catriona A. Ford, Dana Koludrovic, Patricia P. Centeno, Mona Foth, Elpida Tsonou, Nikola Vlahov, Nathalie Sphyris, Kathryn Gilroy, Courtney Bull, Colin Nixon, Bryan Serrels, Alison F. Munro, John C. Dawson, Neil O. Carragher, Valeria Pavet, David C. Hornigold, Philip D. Dunne, Julian Downward, Heidi C.E. Welch, Simon T. Barry, Owen J. Sansom, Andrew D. Campbell

**Affiliations:** 1 Cancer Research UK Scotland Institute, Glasgow, United Kingdom.; 2 Huntsman Cancer Institute, University of Utah Health Sciences Center, Salt Lake City, Utah.; 3 Signalling Programme, Babraham Institute, Cambridge, United Kingdom.; 4 Biopharmaceuticals R&D, AstraZeneca, Cambridge, United Kingdom.; 5 The Patrick G. Johnston Centre for Cancer Research, Queen’s University, Belfast, United Kingdom.; 6 Cancer Research UK Scotland Centre, Institute of Genetics and Cancer, University of Edinburgh, Edinburgh, United Kingdom.; 7 Oncogene Biology Laboratory, The Francis Crick Institute, London, United Kingdom.; 8 Bioscience, Early Oncology, AstraZeneca, Cambridge, United Kingdom.; 9 School of Cancer Sciences, University of Glasgow, United Kingdom.

## Abstract

Metastatic melanoma remains a major clinical challenge. Large-scale genomic sequencing of melanoma has identified *bona fide* activating mutations in *RAC1*, which are associated with resistance to BRAF-targeting therapies. Targeting the RAC1-GTPase pathway, including the upstream activator PREX2 and the downstream effector PI3Kβ, could be a potential strategy for overcoming therapeutic resistance, limiting melanoma recurrence, and suppressing metastatic progression. Here, we used genetically engineered mouse models and patient-derived BRAF^V600E^-driven melanoma cell lines to dissect the role of PREX2 in melanomagenesis and response to therapy. Although PREX2 was dispensable for the initiation and progression of melanoma, its loss conferred sensitivity to clinically relevant therapeutics targeting the MAPK pathway. Importantly, genetic and pharmacologic targeting of PI3Kβ phenocopied PREX2 deficiency, sensitizing model systems to therapy. These data reveal a druggable PREX2/RAC1/PI3Kβ signaling axis in *BRAF*-mutant melanoma that could be exploited clinically.

**Significance:**

Cotargeting the MAPK and the PREX2/RAC1/PI3Kβ pathways has remarkable efficacy and outperforms monotherapy MAPK inhibition in *BRAF*-mutant melanoma, supporting the potential of this combination therapy for treating metastatic melanoma.

## Introduction

Despite the development of efficacious targeted therapeutic approaches, resulting in improved overall survival rates over the last 20 years, metastatic melanoma remains a clinical problem. Although surgery is often curative in early-stage disease, the prognosis for patients diagnosed with metastatic melanoma remains poor, due to the ineffectiveness of surgery in disseminated disease alongside rapidly acquired resistance to targeted therapies. Improved understanding of the molecular basis of melanoma, and of both the response and resistance to targeted therapy, remains critical.

Key to success of targeted therapeutics for melanoma are two concurrent, yet independent, approaches—effective targeting of tumor cell–intrinsic driver mutations and their effector pathways, and reversal of tumor-driven immune suppression. Mutations in *BRAF* occur in ∼50% of melanomas, with the vast majority introducing a phosphomimetic V600E substitution (*BRAF*V600E) that confers constitutive kinase activity (1). Although these oncogenic mutations commonly occur in benign melanocytic precursor lesions (naevi), accumulation of oncogenic mutations or loss of tumor-suppressor genes, such as *CDKN2A*, *PTEN*, or *TP53,* drives progression to melanoma. Indeed *BRAF* mutations frequently co-occur with inactivation of PTEN in melanomas (2, 3), eliciting activation of the proproliferative MAPK pathway and PI3K/AKT/mTOR signaling, respectively. PTEN deficiency cooperates with BRAFV600E, contributing to melanomagenesis (4), resistance to BRAF inhibition (4), and metastasis (5). Melanoma also typically harbors a mutation signature driven by UV radiation exposure (6), although this is absent in ∼15% of cases (7).

**Figure 1.**
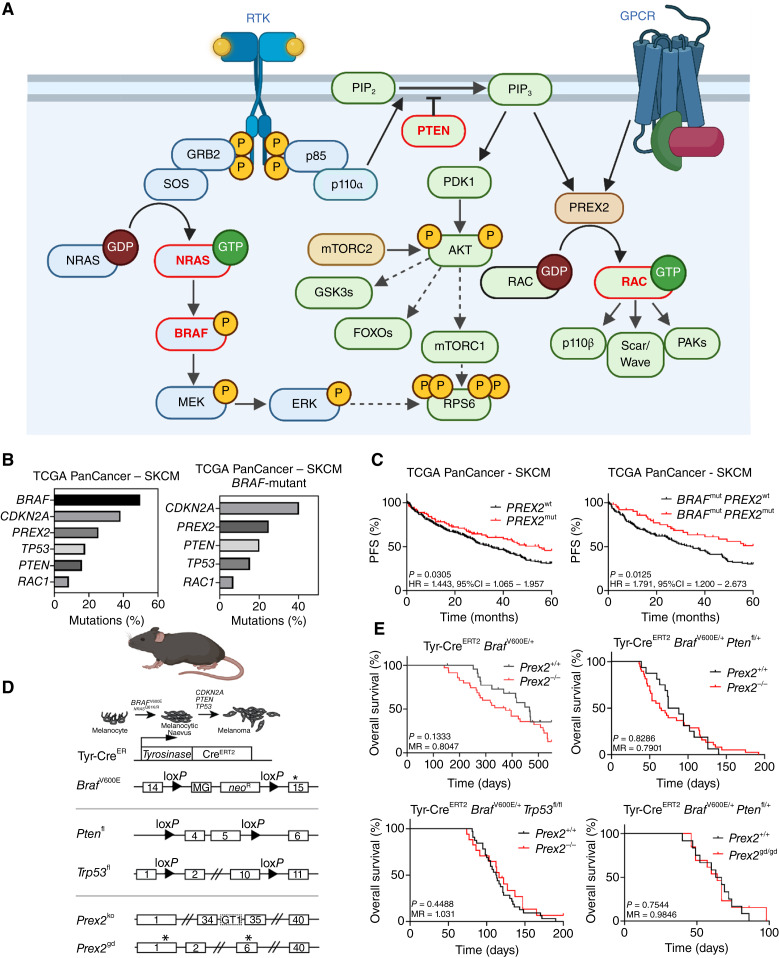
PREX2 ablation does not impact melanoma progression *in vivo*. **A,** Schematic of RTK and G-protein–coupled receptor (GPCR) signaling in melanoma. Arrows, activation; blunt-ended lines, inhibition; circled P, phosphorylation. **B,** Histograms of mutation frequencies of select commonly mutated genes in total (left) and *BRAF*-mutant (right) skin cutaneous melanoma (SKCM) cohorts from TCGA PanCancer study, accessed via cBioPortal on June 23, 2023. **C,** Progression-free survival (PFS) of *PREX2* mutant vs. wild-type cases in the curated cohort of patients with SKCM from the TCGA PanCancer cohort, censored at 5 years. Left, all cases (*n* = 381; *PREX2* wild-type = 290; *PREX2* mutant = 91); right, *BRAF*-mutant cases (*n* = 207; *PREX2* wild-type = 155; *PREX2* mutant = 52). **D,** Top, schematic timeline of melanoma development (melanocytic dysplasia, naevus formation, and progression to melanoma) with common clinically relevant mutations indicated. Bottom, schematics of transgenic alleles used to generate melanoma GEMMs. Exons are boxed, with exon numbers indicated. Asterisks, location of mutations. Filled triangles, loxP sites; MG, minigene encoding exons 15–18 of wild-type *Braf* with a 5′ splice acceptor; neo^R^, neomycin phosphotransferase gene; GT1, gene trap vector containing the β-galactosidase/neomycin resistance fusion gene (β-geo); *Prex2*^gd^, *Prex2*^E22A,N204A^ allele. **E,** Top left, overall survival of Tyr-Cre^ERT2^*Braf*^V600E/+^*Prex2*^+/+^ (*n* = 23; median survival, 466 days) vs. Tyr-Cre^ERT2^*Braf*^V600E/+^*Prex2*^−/−^ (*n* = 35; median survival, 375 days) mice; *P* = 0.1333. Top right, overall survival of Tyr-Cre^ERT2^*Braf*^V600E/+^*Pten*^fl/+^*Prex2*^+/+^ (*n* = 16; median survival, 81 days) vs. Tyr-Cre^ERT2^*Braf*^V600E/+^*Pten*^fl/+^*Prex2*^−/−^ (*n* = 38; median survival, 64 days) mice, *P* = 0.8286. Bottom left, overall survival of Tyr-Cre^ERT2^*Braf*^V600E/+^*Trp53*^fl/fl^*Prex2*^+/+^ (*n* = 32; median survival, 112.5 days) vs. Tyr-Cre^ERT2^*Braf*^V600E/+^*Trp53*^fl/fl^*Prex2*^−/−^ (*n* = 17; median survival, 116 days) mice, *P* = 0.4488. Bottom right, overall survival of Tyr-Cre^ERT2^*Braf*^V600E/+^*Pten*^fl/+^*Prex2*^+/+^ (*n* = 12; median survival, 65 days) vs. Tyr-Cre^ERT2^*Braf*^V600E/+^*Pten*^fl/+^*Prex2*^gd/gd^ (*n* = 13; median survival, 64 days) mice, *P* = 0.7544. *P* values calculated using the log-rank (Mantel–Cox) test. PIP_2_, phosphatidylinositol-4,5-diphosphate; PIP_3_, phosphatidylinositol-3,4,5-trisphosphate; RTK, receptor tyrosine kinase. [**A** and **D,** Created in BioRender. Campbell, A. (2025), https://BioRender.com/n12n755.]

The prevalence of *BRAF* mutations, paucity of effective clinical approaches, and poor prognosis made *BRAF*-mutant metastatic disease ideal for adoption of novel small molecules such as vemurafenib, selectively targeting the BRAFV600E oncoprotein (8). Although targeted BRAF inhibition with vemurafenib or dabrafenib, or combined BRAF and MEK1/2 inhibitors (vemurafenib/trametinib, dabrafenib/cobimetinib, or encorafenib/binimetinib) are now mainstays metastatic melanoma treatment, therapeutic resistance poses a significant clinical challenge (9), and calls for new strategies targeting therapeutic resistance, melanoma recurrence, and metastatic progression (Fig. 1A).

Our search for new therapeutic approaches has focused upon the RAC1-GTPase signaling pathway. Integral to this pathway are guanine nucleotide exchange factors (GEF), such as the phosphatidylinositol-3,4,5-trisphosphate–dependent RAC exchangers PREX1 and PREX2, which promote the conversion of RAC1 from an inactive GDP-bound form to an active GTP-bound conformation (10, 11). This switch potentiates binding of downstream effectors, such as the catalytic PI3K isoform p110β (encoded by *PIK3CB*), which dictate the output of RAC1-driven signaling (12). We have demonstrated that the RAC1-GEF PREX1 plays critical roles in melanoblast migration during early murine embryonic development, melanoma cell invasion and migration, and metastasis in a patient-relevant preclinical model of *NRAS*-mutant melanoma (13). Similarly, we demonstrated that RAC1 is required for embryonic melanoblast migration and function, melanoma growth *in vivo*, and tumor-cell invasion and migration (Fig. 1A; refs. 14, 15).

Although overexpression in melanoma can elicit invasion and metastasis, *PREX1* is rarely mutated in human cancer (13, 16). However, *bona fide* tumor-associated activating mutations in *RAC1* have been identified (17, 18), with the *RAC1*P29S hotspot mutation associated with resistance to BRAF-targeted therapies (19, 20). In addition, rearrangements, amplifications, missense, and nonsense/truncating mutations in the RAC-GEF *PREX2* are frequently detected (21). Although some studies have indicated that truncating mutations may drive constitutive RAC-GEF activity (22, 23), the impact of *PREX2* mutation in melanoma remains contentious.

The tumor suppressor PTEN dephosphorylates phosphatidylinositol-3,4,5-trisphosphate to generate phosphatidylinositol-4,5-diphosphate, antagonizing PREX2 activity (24) and PI3K/AKT signaling. PTEN can also directly inhibit the GEF activity of PREX2 toward RAC1, suppressing cell migration and invasion (24). The corollary of these findings is that *PTEN* loss would upregulate the GEF activity of PREX2 and, consequently, stimulate RAC1 function, potentially rendering targeting of PREX2 in the context of PTEN-deficient melanoma a rational approach. Intriguingly, melanoma-associated RAC1-activating mutations seem to be mutually exclusive with *PTEN* loss, suggesting a scenario whereby loss of *PTEN* drives wild-type RAC1 activation via PREX2 activation (24, 25). Conversely, PREX2 can inhibit PTEN, promoting cell proliferation and tumorigenesis by activating downstream PI3K/AKT signaling (Fig. 1A; refs. 23, 25, 26).

Building on these observations, we use genetically engineered mouse models (GEMM) and xenografts to characterize the role of PREX2 and p110β in BRAFV600E*-*driven melanoma *in vivo*. Moreover, we assess how inactivating mutation of *Prex2* or *Pik3cb *impacts the therapeutic efficacy of MAPK-targeting agents in a preclinical setting. Our data suggest that although inactivation of either *Prex2* or *Pik3cb* has little impact upon tumor initiation or progression, both sensitize to MEK1/2 inhibition. Moreover, we demonstrate that inactivation of *Prex2* abrogates the therapeutic impact of *p110β* inhibition in combination with MEK1/2 inhibition, suggesting an epistatic relationship between the two. In summary, our studies indicate that cotargeting of the MAPK and PREX2/RAC1/p110β signaling axes may be an efficacious therapeutic strategy in BRAFV600E-driven melanoma.

## Materials and Methods

### Mouse studies

All mouse studies were performed in accordance with UK Home Office regulations, under project licenses 70/8646 and PP3908577, and were approved by the Animal Welfare and Ethical Review Board of the University of Glasgow. All mice were housed in conventional cages within a dedicated animal facility at a constant temperature (19–23°C) and humidity (55% ± 10%), with a 12-hour light/dark cycle and *ad libitum* access to food and water. The transgenic alleles used is this study were Tyr-Cre^ERT2^ (27), *Braf*^LSL-V600E^ (28), *Pten*^fl^ (29), *Trp53*^fl^ (30), *Prex2*^ko^ (31), *Prex2*^E22A,N204A^ (designated *Prex2*^gd^) and *Pik3cb*^S205D,K224A^ (designated *Pik3cb*^rbd^; ref. 12). All animal cohorts were maintained as C57Bl6 strains, typically inbred to a minimum of four generations. Genotyping for transgenic alleles and genetic background testing was carried out by Transnetyx. For all mouse studies, no formal randomization was performed but researchers were blinded to the mouse genotypes for analysis.

For melanoma GEMMs, genetic recombination was induced through daily topical administration of 2 mg tamoxifen (T5648, Sigma-Aldrich) on five consecutive days in mice of both sexes, ages 6 weeks to 6 months. Mice were monitored by visual inspection for the development of melanocytic naevi and cutaneous melanoma at least twice weekly, with melanoma volume calculated as *V* = (length × width^2^)/2, in which length is the greatest longitudinal diameter and width is the greatest transverse diameter, as measured by calipers. Tumor-bearing mice were sampled when exhibiting a cutaneous melanoma ≥15 mm in diameter, or tumor ulceration.

For xenograft studies, 1 × 10^6^ WM266.4 or A375 cells or their derivatives were resuspended in 100 µL of sterile PBS and subcutaneously engrafted into the flank of athymic CD1-Foxn1^nu^ mice (Charles River). Tumor outgrowth was monitored by caliper measurements over time. Mice were aged to a humane endpoint defined by tumor size (diameter ≥15 mm) or ulceration.

### Generation of the Prex2^gd^ mouse strain

To generate a mouse strain with catalytically inactive (GEF-dead) PREX2, residues Glu^22^ and Asn^204^ in the catalytic DH domain were mutated to alanine using CRISPR/Cas9 gene editing. First, 20-nt single-guide RNAs (sgRNA) were designed to direct wild-type Cas9 to the relevant sites in exons 1 and 6 of *Prex2*, to be positioned directly upstream of a requisite 5′-NGG protospacer adjacent motif (PAM), and to have no highly homologous sites elsewhere in the mouse genome. Off-target potential was scored using software from the Feng Zhang laboratory (http://crispr.mit.edu/), Broad Institute (http://www.broadinstitute.org/rnai/public/analysis-tools/sgrna-design), and E-CRISPR (http://www.e-crisp.org/E-CRISP/). Three sgRNAs were selected for each target site. For exon 1, they were sgRNA 1 (GCG​CTG​AGC​ACG​CAC​ACG​CGC-*AGG*), sgRNA 2 (GAA​GAC​CGA​GCG​CGA​CTA​CG-*TGG*), and sgRNA 3 (GCG​CGA​CTA​CGT​GGG​CAC​GC-*TGG*), with the adjacent PAM sequence in italics. For exon 6, they were sgRNA 4 (GCG​TGT​GTT​CCA​ACA​TTA​ATG-*AGG*), sgRNA 5 (GTT​GGA​ACA​CAC​GGC​TTT​CA-*TGG*) and sgRNA 6 (GTA​ATG​AGG​CCA​AGA​GAC​AGA-*TGG*). sgRNAs efficiency was assessed *in vitro* using the sgRNA *In Vitro* Transcription and Screening kit (Clontech, 631439) following the manufacturer’s protocol, with 2 kb templates containing the target sequence amplified from mouse genomic DNA using primers GGT​CAG​TGG​TGT​GGT​TGT​TT and CCA​CCA​AGT​CCA​GCT​CAA​AT for exon 1, and TCA​GTT​TTG​AAA​TTG​TGG​TGC​A and GCT​GAG​GGA​CAT​TCA​AGA​CC for exon 6, respectively. All sgRNAs directed efficient cutting of the DNA by Cas9, which produced DNA fragments of the expected sizes. Two sgRNAs for each exon, namely sgRNAs 2 and 3 for exon 1, and sgRNAs 4 and 6 for exon 6, were selected for further assessment. Their efficacy in cells was tested using the Surveyor Mutation Detection Kit (Integrated DNA Technologies) following the manufacturer’s instructions. NIH/3T3 cells were transfected with pSpCas9(BB)-2A-GFP carrying the selected sgRNAs; genomic DNA was extracted, the relevant region amplified, annealed with wild-type DNA, and treated with Surveyor nuclease to cut at the mismatches. Following that, sgRNA 3 was chosen for targeting exon 1 and sgRNA 6 for exon 6.

A total of 200 bp ssDNA repair templates were designed to introduce the desired point mutation by homology-directed repair, to introduce silent mutations creating restriction sites for screening purposes and, where possible, destroy the PAM sites, and to have symmetric homology arms of ≥90 nt. The exon 1 repair template was CTT​GCC​CCC​AGC​TCC​GCG​CCC​CGC​CGG​CCA​CCA​TGA​GCG​ACG​AAA​GCG​CCA​GGG​AAG​TAG​ACA​AGC​AGC​TTC​GCC​TGC​GCG​TGT​GCG​TGC​TCA​GC**GCT**CTt​CAG​AAG​ACC​GAG​CGC​GAC​TAC​GTG​GGt​ACc​CTa​GAG​TTC​CTG​GTG​TCG​GTG​AGT​AGC​CGG​CCC​CGC​GCA​CGG​CAC​CAA​GTC​TGG​AGC​ATT​GTC​TGC (nts coding for the E22A mutation in bold, silent mutations in lower case). The exon 6 repair template was TCT​CCA​CAG​GAA​TTA​CTG​AAG​CGG​ACT​CCA​CGG​AGA​CAT​AGT​GAC​TAC​ACA​GCA​GTG​ATG​GAA​GCA​CTC​CAA​GCC​ATG​AAA​GCC​GTG​TGT​TCC​AAt​ATT**GCT**GAG​GCC​AAG​cGg​CAa​ATG​GAG​AAA​CTG​GAA​GTT​TTA​GAA​GAG​TGG​CAG​GCA​CAC​ATT​GAA​GGC​TGG​GAG​GTA​CGT​GTC​CTT​TGC​TCA​GCT​CTT (nts coding for the N204A mutation in bold, silent mutations in lower case). These ssDNAs were purchased from Dharmacon as PAGE-purified Ultramer ssDNAs.

The selected sgRNA, ssDNA repair template, and Cas9 mRNA were microinjected into the pronucleus of C57BL/6J mouse zygotes by the Babraham Gene Targeting Facility. Initially, mice carrying the E22A mutation were generated. Pups were genotyped by sequencing of 571 bp PCR products amplified from genomic DNA using GAC​TGT​CCC​GTT​CTG​AGT​CC forward and AAT​TTG​CCC​TGG​GAG​ATG​GA reverse primers. Heterozygous *Prex2*^E22A/+^ mice were bred together to generate homozygous *Prex2*^E22A/E22A^ animals, which were then subjected to a further round of pronuclear injections to target the second site, *Prex2*^N204A^. Pups were genotyped for the N204A mutation by sequencing of 536 bp PCR products, amplified from genomic DNA using TGC​TCA​CTC​ATG​GAT​TTG​ACC forward and TCC​ATC​ACA​CAT​GTC​TCA​GGT reverse primers. *Prex2*^E22A/E22A;N204A/+^ mice were bred together to generate homozygous double knock-in *Prex2*^gd^ (*Prex2*^E22A/E22A;N204A/N204A^) mice, which were born at the expected Mendelian rate, were fertile, bred well, and seemed healthy. Once the *Prex2*^gd^ strain was fully established, routine genotyping was done by Transnetyx.

### *In vivo* therapeutic studies

Clinical-grade AZD6244 (32), AZD8186 (33), AZD8835 (34), AZD2014 (35), and AZD5363 (36) were supplied by AstraZeneca under a collaborative research agreement. Cobimetinib (#C-1100) and dabrafenib (#D-5678) were supplied by LC Laboratories. For *in vivo* studies, AZD6244 and AZD8186 were prepared in a vehicle of 0.5% (w/v) hydroxypropyl methylcellulose (#09963, Sigma-Aldrich) and 0.1% (v/v) Tween-80 (#P8192, Sigma-Aldrich) in water, and administered at 25 and 50 mg kg^−1^ twice daily, respectively, in 100 μL. Dabrafenib was prepared in a vehicle of 0.5% (w/v) hydroxypropyl methylcellulose and 0.1% (v/v) Tween-80 in water, and administered at a dose of 30 mg kg^−1^, once daily. Cobimetinib was prepared in a vehicle of 2.5% (v/v) DMSO, 0.5% (w/v) hydroxypropyl methylcellulose, and 0.1% (v/v) Tween-80 in water and administered at a dose of 5 mg kg^−1^ once daily. For combination-dosing studies, compounds were co-formulated in the same vehicle and administered with the same dose and volume as monotherapy. For cutaneous melanoma and xenografted tumor treatment studies, mice were enrolled onto vehicle, mono- or combination treatment in a pseudo-randomized manner when exhibiting a cutaneous melanoma >7 mm in diameter. Mice were excluded from study where tumor ulceration was observed within 7 days of enrollment. Therapeutic response was measured both in terms of primary tumor size (growth/regression) and overall survival.

### Transcriptional profiling by RNA sequencing

Melanoma fragments isolated from BRAF PTEN donor mice were maintained as xenografts via subcutaneous implantation into the flank of adult athymic CD1-Foxn1^nu^. P1 tumor fragments were then implanted subcutaneously into the flank of C57Bl6/J recipients, with these recipient mice were enrolled onto treatment when exhibiting a single tumor with a diameter >7 mm. Therapeutic treatments were as outlined in the *in vivo* therapeutic studies section above but sacrificed following 3 days of treatment.

RNA was isolated using the Qiagen RNAeasy mini kit (#74104), in accordance with the manufacturer’s instructions. Tumor tissue was lysed using a Precellys Lysing Kit (#KT03961-1-003-2) and Precellys Evolution tissue homogenizer from Bertin Technologies. RNA quality was assessed using an Agilent 2200 TapeStation, with RNA screen tape. Libraries for cluster generation and DNA sequencing were prepared using the Illumina TruSeq RNA LT Kit. DNA library quantity and quality was assessed on an Agilent 2200 TapeStation (D1000 ScreenTape) and Qubit (Thermo Fisher Scientific), respectively. Libraries were sequenced using the Illumina Next Seq 500. Gene set enrichment analysis was employed using fgsea R package (v1.21.0), using a ranked gene list via limma R package (v3.50.3) in a grouped pairwise manner. Statistical significance was measured with Benjamini–Hochberg FDR <0.05, and normalized enrichment score indicates the upregulation (positive value) and downregulation (negative value).

### Histochemical and IHC staining

Hematoxylin and eosin (H&E) and IHC were performed on 4-µm formalin-fixed paraffin-embedded sections that had previously been baked at 60°C for 2 hours. Staining was performed in a Leica Biosystems BOND RX Autostainer, using antibodies against cyclin D1 (#55506, Cell Signaling Technology), phospho-RPS6 Ser240/Ser244 (#5364, Cell Signaling), and p21 (#ab107099, Abcam). Formalin-fixed paraffin-embedded sections were deparaffinized using BOND Dewax solution (#AR9222, Leica Biosystems) and epitope retrieval using ER2 solution (#AR9640, Leica Biosystems) for 20 minutes at 95°C, except for cyclin D1, where sections underwent retrieval for 30 minutes. Sections were rinsed with BOND Wash Solution (#AR9590, Leica Biosystems) before endogenous peroxidase blocking using a BOND Intense R Detection kit (#DS9263, Leica Biomarkers) for 5 minutes. Typically, for IHC, sections were rinsed with Wash Solution before application of a blocking solution from an anti-rat ImmPRESS detection kit (#MP7444-15, Vector Laboratories) for 20 minutes. Sections were then rinsed with Wash Solution before application of primary antibodies at an optimized dilution (cyclin D1, 1:150; Ki67, 1:1000; RPS6 Ser240/Ser244, 1:1000; p21, 1:250) for 30 minutes. Sections were then rinsed with Wash Solution and incubated for 30 minutes with anti-rabbit EnVision^+^ horseradish peroxidase–labeled polymer secondary antibody (K4003, Agilent), except for p21 where an anti-rat ImmPRESS secondary antibody was applied. Sections were rinsed with wash buffer and visualized using 3,3′-diaminobenzidine from the BOND Intense R Detection kit.

IHC staining for phospho-RPS6 Ser235/Ser236 (#4858, Cell Signaling Technology) and phospho-p44/42 MAPK (ERK1/2; ERK1, Thr202/Tyr204/ERK2,Thr185/Tyr187; #9101, Cell Signaling Technology) was performed on an Agilent Autostainer Link 48. Sections were loaded into an Agilent pre-treatment module, deparaffinized and subjected to heat-induced epitope retrieval, at 97°C for 20 minutes, using EnVision FLEX target retrieval solution, high pH (#K8004, Agilent). After heat-induced epitope retrieval, sections were washed thoroughly with EnVision FLEX Wash Buffer (#K8007, Agilent), loaded onto the autostainer, subjected to endogenous peroxidase blocking (#S2023, Agilent) for 5 minutes, and rinsed with FLEX Wash Buffer. Primary antibodies were applied at an optimized dilution (phospho-RPS6 Ser235/Ser236, 1:75; phospho-p44/42 MAPK, ERK1/2, 1:400) for 30 minutes at room temperature. Sections were then rinsed with FLEX Wash Buffer, incubated with anti-rabbit EnVision secondary antibody for 30 minutes, and rinsed with FLEX Wash Buffer. Staining was visualized with Liquid DAB^+^ (#K3468, Agilent). After staining for 10 minutes, sections were washed in tap water and counterstained with hematoxylin “Z” stain (#RBA-4201-00A, CellPath).

H&E staining was performed with a Leica Autostainer (#ST5020). Sections were dewaxed, rehydrated through graded alcohols, stained with hematoxylin “Z” stain (#RBA-4201-00A, CellPath) for 13 minutes, washed in tap water, differentiated in 1% (v/v) acid alcohol, washed, with nuclei blued in Scott’s tap water substitute. After further washing, the sections were stained with Putt’s Eosin for 3 minutes.

After H&E staining or IHC, sections were rinsed in tap water, dehydrated through graded ethanol, and placed in xylene. The stained sections were coverslipped in xylene using DPX mountant (#SEA-1300-00A, CellPath).

### Cell culture

WM266.4, A375, WM793, WM1158, and A2058 cells were the kind gift of Prof. Lionel Larue (Institut Curie). UACC-62 cells were purchased from Caltag Medsystems (#C0020003), and SK-MEL-28 cells were purchased from LGC Standards (#HTB-72). Cell line authentication was carried out using the GenePrint 10 System (#B9510, Promega), and lines confirmed free from *Mycoplasma* infection by the CRUK Scotland Institute Molecular Technologies service. WM266.4, A375, WM1158, A2058, and their derivatives were maintained in DMEM (#21969, Thermo Fisher Scientific) supplemented with 10% (v/v) FCS (#10270, Thermo Fisher Scientific), 200 µmol/L L-glutamine (#25030, Thermo Fisher Scientific), and 100 U/mL^−1^ penicillin/streptomycin (#15140, Thermo Fisher Scientific). WM793 and UACC-62 were maintained in RPMI 1640 (#31870, Thermo Fisher Scientific) medium supplemented with 10% (v/v) FCS, 200 µmol/L L-glutamine, and 100 U/mL^−1^ penicillin/streptomycin (#15140, Thermo Fisher Scientific). SK-MEL-28 cells were maintained in Minimum Essential Medium (#11095080, Thermo Fisher Scientific) medium, supplemented with 10% (v/v) FCS, 200 µmol/L L-glutamine, 1% non-essential amino acids (#11140035, Thermo Fisher Scientific), 1 mmol/L sodium pyruvate (#11360070, Thermo Fisher Scientific), and 100 U/mL^−1^ penicillin/streptomycin (#15140, Thermo Fisher Scientific). Cells were harvested by trypsinization with 0.25% (w/v) trypsin (#15090, Thermo Fisher Scientific), washed with PBS, and centrifuged. Cell pellets were resuspended in the appropriate culture medium. The enzymatic activity of trypsin was blocked by resuspension in complete medium, and cell suspensions were centrifuged, washed in PBS, and resuspended in the appropriate culture medium.

For *in vitro* drug treatments, all inhibitors were prepared in DMSO (Sigma-Aldrich) as a 1,000× stock and diluted into the appropriate buffer/medium to the indicated final concentration – AZD6244, 100 nmol/L; AZD8186, 250 nmol/L; AZD8835, 250 nmol/L; AZD2014, 500 nmol/L; AZD5363, 250 nmol/L, TGX-221, 100 nmol/L (#HY-10114, MedChemTronica); AZD6482, 100 nmol/L (#HY-10344, MedChemTronica); SAR-260301, 250 nmol/L (#HY-15837, MedChemTronica); cobimetinib, 30 nmol/L, and dabrafenib, 30 nmol/L.

### Cell proliferation

WM266.4 (2 × 10^3^), A375 (1.3 × 10^3^), or WM793 (1.5 × 10^3^) cells were seeded into 96-well plates for 24 hours before addition of AZD6244 (100 nmol/L), AZD8186 (250 nmol/L), AZD8835 (250 nmol/L), AZD2014 (500 nmol/L), AZD5363 (250 nmol/L), TGX-221 (100 nmol/L), AZD6482 (100 nmol/L), SAR-260301 (250 nmol/L), cobimetinib (30 nmol/L), dabrafenib (30 nmol/L), a combination of these drugs, or DMSO for up to 96 hours. Growth of cultures was monitored at 6-hour intervals for up to 72 or 96 hours using an IncuCyte live-cell analysis system (Essen Bioscience). IncuCyte software was used to measure relative cell confluence, which was normalized to starting confluence. Experiments were performed independently at least three times with technical triplicates.

### Immunoblotting

WM266.4, A375, or WM793 cells were seeded (2 × 10^5^ cells) in media (DMEM for WM266.4 and A375 and RPMI for WM793) supplemented with 10% FBS, 2 mmol/L glutamine +100 U mL^−1^ penicillin/streptomycin, into six-well plates for 24 hours before culturing in AZD6244 (100 nmol/L), AZD8186 (250 nmol/L), AZD8835 (250 nmol/L), AZD2014 (500 nmol/L), AZD5363 (250 nmol/L), or a combination of these drugs for 24 hours. Cells were harvested and lysed using RIPA with protease inhibitors (Roche 11836153001) and phosphatase inhibitors (Roche 04906845001). Protein (20 μg) was loaded onto a 4% to 12% bis-tris gradient protein gel (Invitrogen) under reducing conditions. For enhanced chemiluminescence imaging, resolved proteins were transferred onto the polyvinylidene difluoride membrane. Membranes were blocked in 5% milk/TBST, probed overnight with primary antibodies diluted in 5% BSA/TBST followed by suitable horseradish peroxidase–conjugated secondary antibodies diluted in 5% milk/TBST. For fluorescence-based imaging, proteins were transferred onto a nitrocellulose membrane (Bio-Rad, #170-4270), using the Bio-Rad Trans-Blot Turbo Transfer System. Membranes were blocked in 5% BSA (Sigma-Aldrich, #A7906)/TBST, probed overnight with primary antibodies diluted in 5% BSA/TBST, followed by fluorescently conjugated secondary antibodies (anti-rabbit IgG, Thermo Fisher Scientific, cat. #A32735; anti-mouse IgG, Thermo Fisher Scientific, cat. #A21057). Primary antibodies from Cell Signaling Technology were phospho-Akt (Ser473) 3787, pan-Akt 2920, phospho-Erk1/2 (Thr202/Tyr204) 9101, total-Erk1/2 9107, phospho-S6 ribosomal protein (Ser235/Ser236) 2211, phospho-S6 ribosomal protein (Ser240/Ser244) 5364, total-S6 ribosomal protein 2317, phospho-4E-BP1 (Thr37/Thr40) 2855, phospho-Rb (Ser793) 9301, total-Rb 9309, and cyclin D1 2978. The antibodies against total 4E-BP1 and β-actin were from Novus (NBP1-47366) and Sigma-Aldrich (A2228), respectively. ECL blots were developed using Clarity Western ECL and ChemiDoc Imaging System (Bio-Rad). Fluorescence imaging was performed with the LI-COR Odyssey CLx Infrared Imaging System.

### Reverse-phase protein array

WM266.4, WM793, WM1158, A375, and A2058 cells treated with AZD6244, AZD8186, AZD8835, or combinations of these for 24 hours were washed with PBS, then lysed in a buffer comprised of 1% Triton X-100, 50 mmol/L HEPES (pH 7.4), 150 mmol/L sodium chloride, 1.5 mmol/L magnesium chloride, 1 mmol/L EGTA, 100 mmol/L sodium fluoride, 10 mmol/L sodium pyrophosphate, 1 mmol/L sodium vanadate, and 10% (v/v) glycerol, supplemented with cOmplete ULTRA and PhosSTOP protease and phosphatase inhibitor cocktails (Roche). Following clearing by centrifugation, lysates were diluted to produce a dilution series of each sample and spotted onto nitrocellulose-coated slides (Grace BioLabs) in triplicate using an Aushon 2470 array platform (Aushon Biosystems). Slides were then blocked in SuperBlock (TBS) blocking buffer (Thermo Fisher Scientific) and incubated with validated primary antibodies (1:250; Supplementary Table S1). Bound antibodies were detected by incubation with DyLight 800–conjugated secondary antibodies (New England BioLabs) and analyzed using an InnoScan 710-IR scanner (Innopsys). The relative fluorescence intensity of each array feature was quantified using Mapix software (Innopsys). Intensity values were normalized to the DMSO control samples for each cell line, and log_2_ transformed data subsequently plotted.

### *In vitro* cell-cycle analysis

WM266.4 or A375 cells were seeded at 4 × 10^5^ cells/6-cm petri dish in DMEM supplemented with 10% (v/v) FBS, 2 mmol/L glutamine, and 100 U/mL^−1^ penicillin/streptomycin. After 24 hours, cells were synchronized by culturing in serum-free medium for a further 24 hours. Synchronized cultures were then treated for 24 hours with AZD6244 (100 nmol/L), AZD8186 (250 nmol/L), a combination of AZD6244 (100 nmol/L) and AZD8186 (250 nmol/L; AstraZeneca), or DMSO, in DMEM containing 10% FBS, 2 mmol/L glutamine, and 100 U/mL^−1^ penicillin/streptomycin. Culture supernatants and trypsinized cells were harvested, and pelleted cells were fixed in ice-cold 70% ethanol. Fixed cells were stained with FxCycle PI/RNase staining solution (#F10797, Thermo Fisher Scientific) as per manufacturer’s instructions and acquired on the Attune Flow Cytometer (Thermo Fisher Scientific) followed by analysis using FlowJo software (BD Biosciences). Experiments were performed independently at least three times.

### CRISPR/Cas9-mediated genome editing

Genome editing was performed in WM266.4 cells. Alt-R S.p. Cas9 Nuclease V3, 100 µg (#1081058), Cas9 Electroporation Enhancer (#1075915), tracrRNA, and guides were purchased from Integrated DNA Technologies and prepared according to the manufacturer’s instructions. Electroporation was performed with the Lonza SF Cell line 4D-Nucleofector X solution (#V4XC-2032) and the 4D-Nucleofector X Unit (Lonza; #AAF-1003X) using program CM137. To validate the CRISPR knockout, gene-edited sequences were PCR-amplified and sequenced (in-house Molecular Technology Service), and knockout efficiency was quantified using the decomposition algorithm, Tracking of Indels by Decomposition (TIDE; ref. 37). Targeting gRNAs were designed by and purchased from Integrated DNA Technologies as follows:

*PREX1* 5′-GCTATACCGTCACCAACGGCTGG-3′
*PREX2* 5′-TCGTGGCCGGATCAACACGGAGG-3′
*PIK3CB* 5′-CTTCCCGAGGTACCTCCAACTGG-3′

Primers used for coding region amplification were as follows:

*PIK3CB* forward: 5′-TCC​TTG​ACA​TCT​GGG​CGG​TGG​A-3′
*PIK3CB* reverse: 5′-AGG​CAA​GCC​TGC​TGA​GGG​AAA​A-3′
*PREX1* forward: 5′-GCC​CAG​GAA​GCA​TTT​TGG​GGC​T-3′
*PREX1* reverse: 5′-TGC​CCC​TTC​CCT​AGA​CAC​AGC​C-3′
*PREX2* forward: 5′-CAG​AGT​CTG​ATT​GGG​CAC​CGC​T-3′
*PREX2* Reverse: 5′-TCA​CAG​TAG​TCC​TCC​CCT​CCC​T-3′.

### Statistical analysis and data visualization

Statistical analysis and graph plotting was carried out using GraphPad Prism (10.0.2). All comparisons made, and statistical tests used are described in the appropriate legend.

### Data availability

All data presented in this article are archived on the CRUK Scotland Institute servers and are available from the corresponding author on request. The transcriptional data generated in this study are publicly available in Gene Expression Omnibus (https://www.ncbi.nlm.nih.gov/geo), with accession number GSE281123. Human clinical data that support the findings of this study are available in cBioPortal (https://www.cbioportal.org) and are in whole or part based upon data generated by The Cancer Genome Atlas (TCGA) Research Network (https://www.cancer.gov/tcga) or as part of the MSK-IMPACT clinical sequencing cohort.

## Results

### Deletion of *Prex2* does not impact development and progression of BRAFV600E-driven melanoma *in vivo*

Although *PREX2* mutations are detected in ∼26% of human melanoma samples (Fig. 1B), these broadly lack functional annotation. Although *PREX2* is most frequently mutated in melanoma, pan-cancer studies have identified mutations in multiple tumor types (Supplementary Fig. S1A and S1B; Supplementary Table S2; ref. 38). The biological and clinical significance of these mutations remains unclear. Biochemical analyses suggest that truncating *PREX2* mutations may have a protumorigenic role (22, 23), although this is confounded by clinical data from TCGA PanCancer dataset, indicating that *PREX2* mutation is associated with extended progression-free survival in a curated cohort of cutaneous melanoma, irrespective of *BRAF* status (Fig. 1C; Supplementary Fig. S1C), but with no improvement in overall survival (Supplementary Fig. S1D and S1E; ref. 7). Notably, total mutation burden seems to be elevated in *PREX2* mutant tumors and associated with improved overall survival (Supplementary Fig. S1F), suggesting that PREX2 mutation may be associated with a favorable mutator phenotype.

Considering this, we tested the functional role of PREX2 in melanoma *in vivo*, combining GEMMs of *BRAF*V600E-mutant melanoma with constitutive *Prex2* deletion. Oncogenic activation of *Braf*^V600E^ and/or deletion of tumor suppressor genes was targeted to the adult melanocyte population with a tamoxifen-inducible Cre-recombinase, under the control of the tyrosinase promoter (Tyr-Cre^ERT2^; Fig. 1D, bottom; ref. 27). The resultant phenotype has been well characterized in the mouse (5), with the classical disease trajectory well understood (Fig. 1D, top).

Melanocyte-specific expression of the BRAFV600E oncoprotein (Tyr-Cre^ERT2^*Braf*^V600E/+^–henceforth BRAF) resulted in robust development of naevi 2 to 6 weeks after induction (median onset, 24 days), with lowly penetrant melanoma observed in ∼40% (9/22) of mice within 1 year (median onset, 403 days; Supplementary Fig. S2A), in line with earlier studies carried out with these alleles (39). Melanoma-bearing animals were aged to a defined endpoint (tumor diameter ≥15 mm, or ulceration), with an overall median survival of 466 days (Fig. 1E). To examine the role of PREX2 in melanoma, we interbred BRAF mice with germline deletion of *Prex2* (Tyr-Cre^ERT2^*Braf*^V600E/+^*Prex2*^−/−^ – henceforth BRAF PREX2). Compared with BRAF mice (median onset, 24 days), naevus formation in BRAF PREX2 mice was delayed (median onset, 37 days), with no impact on tumor initiation (median, 301 vs. 407 days), or overall survival (median, 375 vs. 466 days; Fig. 1E; Supplementary Fig. S2A).

To generate more rapid and robust models of melanoma, we combined *Braf* mutation with targeted deletion of the key tumor suppressor genes *Pten* or *Trp53* – both commonly mutated in patients, and both known to synergize with BRAF-V600E in accelerated melanomagenesis in the mouse (Fig. 1B; refs. 5, 39, 40). Given previous reports of the reciprocal inhibition between PREX2 and PTEN (24, 25), we primarily focused on the impact of *Prex2* deletion in models driven by loss of *Pten*, compared with loss of *Trp53* (Fig. 1E; Supplementary Fig. S2B and S2C). Alongside BRAFV600E, deletion of *Pten* (Tyr-Cre^ERT2^*Braf*^V600E/+^*Pten*^fl/+^– BRAF PTEN) or *Trp53* (Tyr-Cre^ERT2^*Braf*^V600E/+^*Trp53*^fl/fl^ – BRAF P53) did not significantly impact naevus formation (median, 32 and 28 days, respectively, compared with 24 days in BRAF cohort). However, deletion of either accelerated primary tumor development (median, BRAF PTEN, 51 days; BRAF P53, 79 days; BRAF, 407 days; Supplementary Fig. Fig. S2A–S2C), and decreased median overall survival (BRAF PTEN, 81 days; BRAF P53, 113 days; BRAF, 466 days; Fig. 1E). Deletion of *Prex2* did not impact naevus onset, primary tumor formation, or overall survival in either BRAF PTEN or BRAF P53 mice (Fig. 1E; Supplementary Fig. S2B and S2C).

These data indicate that PREX2 function is dispensable for early initiation and progression of BRAF-driven melanoma and do not support the existence of a melanomagenesis-relevant mutual inhibition between PTEN and PREX2 *in vivo* (24, 25).

### Loss-of-function mutation of *PREX2* phenocopies genetic deletion in BRAF PTEN melanoma

Although PREX2 primarily functions as a GEF for the small GTPase RAC1, it also sits at the nexus of multiple complex signaling networks; it is conceivable that *Prex2* deletion may elicit off-target phenotypes. Therefore, to determine whether functional loss phenocopies *Prex2* deletion *in vivo*, we generated an enzymatically inactive, “GEF-dead” mutant PREX2 allele based upon validated functional data from human samples (24). We combined this mutant *Prex2*^E22A,N204A^ (*Prex2*^gd^) allele with our BRAF PTEN melanoma model (Tyr-Cre^ERT2^*Braf*^V600E/+^*Pten*^fl/+^*Prex2*^gd/gd^ – BRAF PTEN PREX2-GD; Supplementary Figs. S2D and S3). In line with PREX2 deficiency, this mutation had no impact upon disease trajectory, with naevus onset, melanoma initiation, and overall survival of BRAF PTEN PREX2-GD animals equivalent to the BRAF PTEN control cohort (Fig. 1E; Supplementary Fig. S2D). Notably, the previously observed acceleration of naevus formation (Supplementary Fig. S2B) was not recapitulated in BRAF PTEN PREX2-GD cohorts (Supplementary Fig. S2D). Given that the PREX2-GD protein solely lacks GEF activity, this may be driven by a RAC1 independent PREX2 function, such as the reported reciprocal inhibitory interaction between PREX2 and PTEN. Indeed, although *Prex2* deletion accelerated naevus onset in the context of heterozygous *Pten* deletion, it had no impact upon naevus formation following *Trp53* deletion (Supplementary Fig. S2B and S2C).

### PREX2 loss of function sensitizes to MEK inhibition in BRAF-driven melanoma

**Figure 2.**
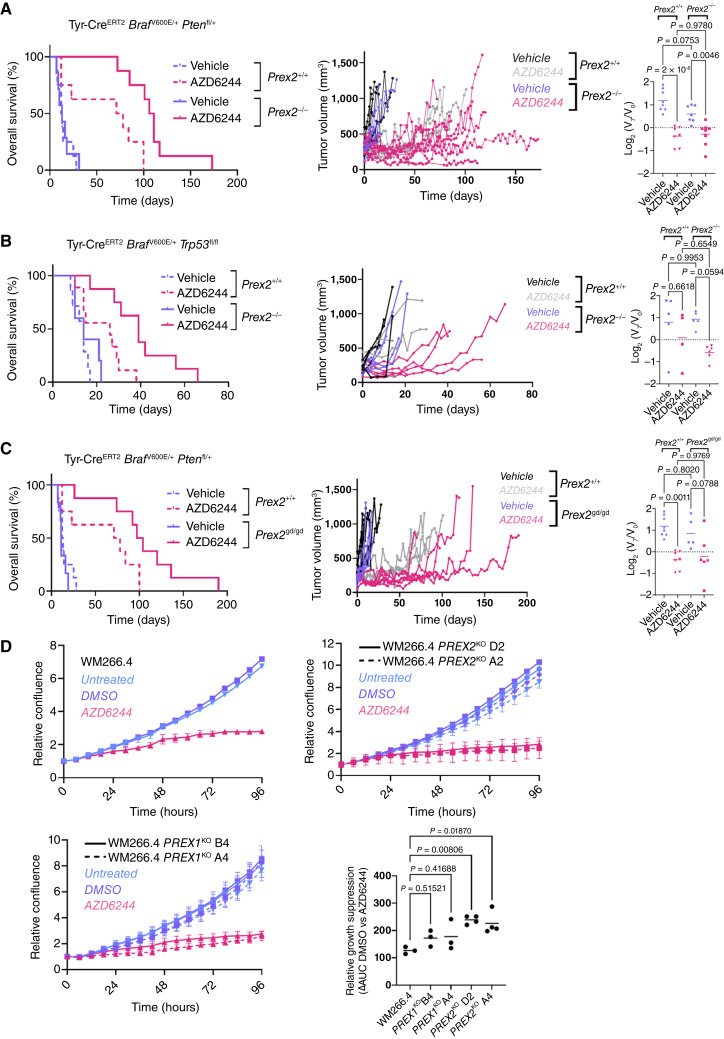
Functional loss of PREX2 function sensitizes to MAPK inhibition *in vitro* and *in vivo*. **A,** Left, Kaplan–Meier overall survival of mice with the indicated genotypes and treatments. BRAF PTEN + vehicle (*n* = 8; median survival, 12.5 days) vs. BRAF PTEN + AZD6244 (*n* = 8; median survival, 74.5 days), *P* = 0.0096; BRAF PTEN PREX2 + vehicle, (*n* = 7; median survival, 13 days) vs. BRAF PTEN PREX2 + AZD6244 (*n* = 8; median survival, 108.5 days), *P* = 0.00005; BRAF PTEN + AZD6244 (*n* = 8; median survival, 74.5 days) vs. BRAF PTEN PREX2 + AZD6244 (*n* = 8; median survival, 108.5 days), *P* = 0.0021 log-rank (Mantel–Cox) test. Middle, longitudinal growth of individual tumors from vehicle-treated (*n* = 8) vs. AZD6244-treated (*n* = 7) BRAF PTEN cohorts and vehicle-treated (*n* = 7) vs. AZD6244-treated (*n* = 8) BRAF PTEN PREX2 cohorts. Right, relative change in tumor volume in mice with the indicated genotypes over the first 7 days of indicated treatment. BRAF PTEN + vehicle (*n* = 8), BRAF PTEN + AZD6244 (*n* = 7), BRAF PTEN PREX2 + vehicle (*n* = 7), BRAF PTEN PREX2 + AZD6244 (*n* = 8). *P* values were calculated by one-way ANOVA corrected for multiple comparisons (Tukey). **B,** Left, Kaplan–Meier overall survival of mice with the indicated genotypes and treatments. BRAF P53 + vehicle (*n* = 7; median survival, 14 days) vs. BRAF P53 + AZD6244 (*n* = 9; median survival, 26 days), *P* = 0.0204; BRAF P53 PREX2 + vehicle (*n* = 5; median survival, 14 days) vs. BRAF P53 PREX2 + AZD6244 (*n* = 8; median survival, 39 days), *P* = 0.0010; BRAF P53 + AZD6244 (*n* = 8; median survival, 14.5 days) vs. BRAF P53 PREX2 + AZD6244 (*n* = 10; median survival, 35 days), *P* = 0.0034. *P* values calculated using the log-rank (Mantel–Cox) test. Middle, longitudinal growth of individual tumors from vehicle-treated (*n* = 5) vs. AZD6244-treated (*n* = 4) BRAF P53 cohorts and vehicle-treated (*n* = 7) vs. AZD6244-treated (*n* = 6) BRAF P53 PREX2 cohorts. Right, relative change in tumor volume over the first 7 days of indicated treatment. BRAF P53 + vehicle (*n* = 5), BRAF P53 + AZD6244 (*n* = 4), BRAF P53 PREX2 + vehicle (*n* = 5), BRAF P53 PREX2 + AZD6244 (*n* = 6); *P* values, one-way ANOVA corrected for multiple comparisons (Tukey). **C,** Left, Kaplan–Meier overall survival of BRAF PTEN and BRAF PTEN PREX2-GD cohorts treated with vehicle or AZD6244. BRAF PTEN + vehicle (*n* = 8; median survival, 12.5 days) vs. BRAF PTEN + AZD6244 (*n* = 8; median survival, 74.5 days), *P* = 0.0096; BRAF PTEN PREX2-GD + vehicle (*n* = 6; median survival, 11 days) vs. BRAF PTEN PREX2-GD + AZD6244 (*n* = 8; median survival, 100.5 days), *P* = 0.0002; BRAF PTEN + AZD6244 (*n* = 8; median survival, 74.5 days) vs. BRAF PTEN PREX2-GD^+^AZD6244 (*n* = 8; median survival, 100.5 days), *P* = 0.0414. *P* values calculated using the log-rank (Mantel–Cox) test. Middle, longitudinal growth of individual tumors from vehicle-treated (*n* = 8) vs. AZD6244-treated (*n* = 7) BRAF PTEN cohorts and vehicle-treated (*n* = 6) vs. AZD6244-treated (*n* = 8) BRAF PTEN PREX2-GD cohorts. Right, relative change in tumor volume over the first 7 days of indicated treatment. BRAF PTEN + vehicle (*n* = 8), BRAF PTEN + AZD6244 (*n* = 7), BRAF PTEN PREX2-GD + vehicle (*n* = 5), BRAF PTEN PREX2-GD + AZD6244 (*n* = 6). *P* values calculated by one-way ANOVA corrected for multiple comparisons (Tukey). Note that the same BRAF PTEN treatment datasets are represented in **A** and **C**. **D,** Top left, relative confluence of parental WM266.4 cells treated with indicated treatments over the 96-hour period. Representative of a minimum of three independent experiments and three technical replicates. Data, mean ± SEM (confluence relative to starting point). Top right, relative confluence of WM266.4 *PREX2*^KO^ pooled lines D2 and A4 treated with indicated treatments over the 96-hour period. Representative of a minimum of four independent experiments and three technical replicates. Data, mean ± SEM (confluence relative to starting point). Bottom left, relative confluence of WM266.4 *PREX1*^KO^ pooled lines B4 and A4 treated with indicated treatments over the 96-hour period. Representative of a minimum of three independent experiments and three technical replicates. Data, mean ± SEM (confluence relative to starting point). Bottom right, relative suppression of growth, difference in calculated AUC of DMSO treated vs. AZD6244-treated lines as indicated. Center line, mean. *P* values calculated by one-way ANOVA corrected for multiple comparisons (Tukey).

Both loss-of-function mutation of PTEN and activating mutation of RAC1, are known to modulate the response to BRAF inhibition (4, 41, 42). We therefore addressed whether PREX2 impacts targeted therapeutic responses. Given that MAPK pathway activation is the key downstream effector of BRAFV600E, and that effective targeting of this pathway is a current standard-of-care for *BRAF*-mutant melanoma, we tested the efficacy of the MEK1/2 inhibitor, selumetinib (AZD6244; ref. 43), in PREX2-deficient versus -proficient BRAF PTEN and BRAF P53 melanoma. AZD6244 had significant efficacy in BRAF PTEN mice, although in BRAF P53, despite short-term suppression of tumor growth, rapid regrowth suggested either intrinsic resistance, or the ability to rapidly reactivate suppressed signaling pathways (Fig. 2A and B). Combined MAPK targeting via cobimetinib (MEK1/2 inhibitor) and dabrafenib (BRAF inhibitor), a clinical approach used to abrogate rapid acquired resistance to MAPK targeting resulted in deeper and more prolonged responses than monotherapy AZD6244 in BRAF PTEN melanoma (Supplementary Fig. S4A). Nonetheless, the modest efficacy of AZD6244 monotherapy *in vivo* provided an effective platform to characterize modifiers of this response. For all treatments, mice were enrolled onto treatment having developed a single melanoma of diameter 7 to 10 mm (Supplementary Fig. S4B) and treated to clinical endpoint.

We next determined whether deletion of *Prex2* impacts the baseline therapeutic efficacy of AZD6244. BRAF PTEN PREX2 and BRAF P53 PREX2 were sensitive to AZD6244, with response resulting in substantial tumor regression in both models, albeit with more prolonged sensitivity in BRAF PTEN PREX2 (Fig. 2A and S2B; Supplementary Fig. S4B). These data indicate that PREX2 may modify the response to MAPK-targeting therapies in BRAF-driven melanoma. We next assessed biomarker expression following short-term treatment with AZD6244, where mice were sampled after 5 days of treatment. MAPK and mTORC1 signaling were equally suppressed by treatment in the presence and absence of *Prex2*, indicated by reduced phosphorylation of ERK1/2 and ribosomal protein S6 (RPS6), at sites phosphorylated by MAPK and mTORC1 signaling respectively (Supplementary Fig. S4C).

As PREX2 deficiency prolonged responses to MAPK-targeted therapy, BRAF PTEN PREX2-GD was used to determine whether this effect was attributable to the RAC-GEF activity of PREX2. BRAF PTEN PREX2-GD mice were enrolled onto AZD6244 or vehicle control and assessed for both immediate and prolonged impact. Treatment of BRAF PTEN PREX2-GD mice with a MAPK inhibitor caused tumor regression and extended overall survival (Fig. 2C). Responses were similar in BRAF PTEN PREX2 and BRAF PTEN PREX2-GD (95.5 vs. 89.5 days, respectively), and significantly greater than BRAF PTEN (62 days; Fig. 2A and C). Together, these data suggest that the canonical GEF activity of PREX2 counteracts MAPK inhibition in BRAF PTEN melanoma.

Next, to both validate response data and develop a more tractable *in vitro* system to understand the role of PREX2 under treatment, we used CRISPR/Cas9-mediated gene editing to disrupt the expression of *PREX2* or its close relative *PREX1* in the PTEN-deficient WM266.4 melanoma line. We hypothesized that *PREX2* disruption would acutely sensitize WM266.4 lines to MEK inhibition with AZD6244. We chose to target *PREX1* as it is highly expressed in WM266.4 cells (13) and is both structurally and functionally homologous to PREX2. Following CRISPR/Cas9 genome editing, the TIDE algorithm (37) indicated that 87.1% (D2) or 64.3% (A4) of *PREX2* transcripts*,* and 81.6% (B4) or 82.6 (A4) of *PREX1* transcripts in the edited cell populations were generated from disrupted genomic sequences in their respective polyclonal cell line pools (Supplementary Fig. S4D). Targeted deletion of either PREX2 or PREX1 had no impact upon basal MAPK or PI3K/mTOR activity, in line with *in vivo* studies (Supplementary Fig. S4E), although live-cell imaging indicated that both PREX2 and PREX1 are dispensable for efficient growth of WM266.4, but that PREX2 (and not PREX1) disruption sensitizes to AZD6244-mediated MAPK inhibition (Fig. 2D).

Collectively, these data implicate RAC1 signaling in the resistance to MAPK-targeted therapy and support the reported role for activating *RAC1*P29S mutations in melanoma resistance to vemurafenib (42). Furthermore, they suggest an opportunity for RAC1 pathway targeting agents in resistance-breaking combination approaches for BRAF-mutant melanoma. Nonetheless, direct therapeutic targeting of small GTPases, such as RAC1, has been viewed as challenging – despite the significant recent success of small molecules targeting oncogenic RAS. An alternative is the targeting of RAC1 effector molecules, such as the PIKK family kinase p110β (*Pik3cb*; ref. 12). p110β/*Pik3cb* is a particularly attractive target in light of the impact of PREX2 loss reported here, as not only does it act as a downstream effector of RAC1 (12) but, akin to PREX2, it is known to be co-regulated by the binding of the Gβγ subunit of G-protein–coupled receptors (44, 45) and moreover, exhibits enriched catalytic activity in PTEN-deficient tumors (46, 47).

### Inhibition of the RAC1 effector p110β inhibition phenocopies PREX2 depletion and potentiates MAPK inhibition in human melanoma cells *in vitro*

**Figure 3.**
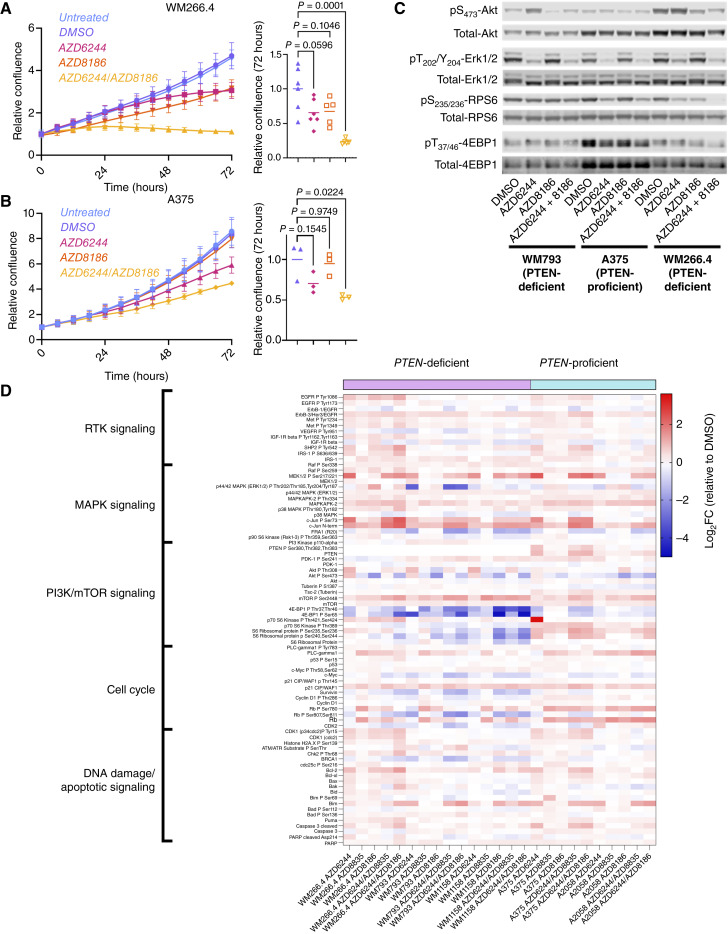
Cotargeting of MEK1/2 and the RAC1 effector p110β suppresses growth of melanoma lines *in vitro*. **A,** Left, relative confluence of WM266.4 cells treated with indicated treatments over time. Representative of a minimum of five independent experiments and three technical replicates. Data, mean ± SEM (confluence relative to starting point). Right, relative change in confluence of WM266.4 cells over indicated 72-hour treatment. Center line, mean. *P* values calculated by one-way ANOVA corrected for multiple comparisons (Tukey). **B,** Left, relative confluence of A375 cells treated with indicated treatments over time. Representative of three independent experiments with a minimum of three technical replicates. Data, mean ± SEM (confluence relative to starting point). Right, relative change in confluence of A375 cells over indicated 72-hour treatment. Center line, mean. *P* values calculated by one-way ANOVA corrected for multiple comparisons (Tukey). **C,** Fluorescence labeled (LI-COR) immunoblotting for indicated activated components of the MAPK–PI3K–mTOR pathway in the indicated human melanoma cells treated with vehicle or the indicated targeted therapeutics. Co-labeled total protein detection for each phosphoprotein serves as a sample integrity control. The blots are representative of three repeated experiments. **D,** RPPA dataset comparing PTEN-deficient and PTEN-proficient melanoma cell lines treated with indicated treatments for 24 hours. Cell line names and treatments are indicated below the heatmap. Antigens, detected by RPPA antibodies, are listed vertically according to biological process/signaling pathway. Color intensity scale indicates high (red) and low (blue) log_2_FC of RPPA intensity values relative to the relevant DMSO control. Results are representative of two technical and five biological replicates per condition.

To validate the RAC1 effector p110β as a potential combination strategy for BRAF-mutant melanoma, we compared the *in vitro* growth kinetics of a *BRAF*-mutant, *PTEN*-deficient melanoma cell line (WM266.4) and a *BRAF*-mutant, *TP53*-deficient but *PTEN*-proficient line (A375) following treatment with AZD8186, a clinically relevant, p110β/δ inhibitor (33). Combined targeting of MEK1/2 and p110β/δ with AZD6244 and AZD8186 almost completely abrogated the growth of PTEN-deficient WM266.4 cells over the same period, in contrast to treatment with AZD6244 alone (Fig. 3A). This combination treatment also elicited a slight, yet significant, suppression of growth of the PTEN-proficient line A375 (Fig. 3B). These observations were supported by subsequent testing of additional independent selective p110β targeting agents, including TGX-221, SAR-260301, and AZD6482. In each case, p110β inhibition potentiated the impact of AZD6244 (Supplementary Fig. S5A and S5B). The impact of p110β targeting was mirrored by combination with more clinically relevant BRAF/MEK targeting strategies. Although combination or monotherapy with cobimetinib (MEK1/2) and dabrafenib (BRAF) was effective in suppressing growth of WM266.4 *in vitro*, this antiproliferative effect was enhanced by combination with AZD8186 (Supplementary Fig. S5C). The impact of combined MEK1/2 and p110β inhibition was most pronounced in the WM266.4 line and ineffective in a further panel of additional human melanoma lines carrying *BRAF* and *PTEN* mutations, namely UACC62, SKMEL28, and WM793 (Supplementary Fig. S6A–S6C). Understanding the determinants of this differential response may be highly informative in future.

Subsequent experiments aimed to better understand the impact of broader PI3K/mTOR pathway inhibition upon growth in the WM266.4 line. The combination of the p110α/δ-selective inhibitor AZD8835 (34) with AZD6244 was marginally more effective than monotherapy AZD6244, but markedly less effective than the combination of AZD8186 and AZD6244. This suggests that in terms of potentiating MAPK inhibition, p110β predominates, although does not preclude p110α activity. Moreover, the effect of AZD6244 was also potentiated by inhibition of the mTOR kinase with AZD2014/vistusertib, suggesting that the observed growth suppression may be mediated suppression of AKT/mTOR signaling downstream of PI3K (Supplementary Fig. S6D and S6E).

To better understand mechanisms underlying therapeutic responses, we assessed their impact upon key signaling nodes from vehicle- and drug-treated melanoma lines – specifically the PTEN-deficient WM266.4 and WM793, and PTEN-proficient A375. These experiments compared responses to AZD6244-mediated MAPK inhibition in combination with inhibitors of p110α (AZD8835), p110β/δ (AZD8186), AKT (AZD5363/capivasertib), or mTOR kinase (AZD2014/vistusertib; Fig. 3C; Supplementary Fig. S7A–S7C).

Monotherapy AZD6244 resulted in suppressed ERK1/2 phosphorylation, with no substantial impact upon PI3K/mTOR signaling. Indeed, *in vitro* time-courses demonstrated that AZD6244 monotherapy suppressed both ERK1/2 and AKT phosphorylation in WM793, A375, and WM266.4 over a period of 1 to 4 hours, with rebound in signaling at 6 to 24 hours (Supplementary Fig. S7D–S7F). This translated into an induction of AKT phosphorylation (Ser473) in PTEN-deficient lines in response to treatment with AZD6244, in a manner sensitive to p110β inhibition/AZD8186 (Fig. 3C), but not to p110α inhibition/AZD8835 (Supplementary Fig. S7A). Similarly, phosphorylation of the mTOR-dependent biomarkers RPS6 (Ser235/236) and 4E-BP1 (Thr37/46) was suppressed in WM266.4 cells in response to combined treatment with AZD6244 and AZD8186 (Fig. 3C), but not in both WM793 and A375 lines, a pattern that correlated with earlier growth response data. This pattern of RPS6/4E-BP1 phosphorylation was conserved in response to combined MAPK/AKT inhibition and seemed resistant to MAPK/p110α inhibition (Supplementary Fig. S7A and S7C). Combined inhibition of MAPK and mTOR kinase efficiently suppressed mTOR effector pathway activity in all cell lines tested (Supplementary Fig. S7B). These data indicate substantial and complex crosstalk/compensation between the MAPK and PI3K/AKT/mTOR pathways imply a role for p110β/δ signaling in WM266.4, and hint that impact upon downstream mTOR signaling may be key to response.

To broaden our understanding of the impact of cotargeting MAPK and PREX2/RAC1/p110β, we performed a targeted proteomics approach via reverse-phase protein array (RPPA). Here, we used a broader collection of established melanoma lines, again encompassing PTEN-deficient (WM266.4, WM793, and WM1158) and PTEN-proficient (A375 and A2058) cells, and sought to better understand any differential role of p110α and p110β through head-to-head comparison of antiproliferative and proapoptotic efficacy of AZD8835 and AZD8186.

We assessed >50 key signaling nodes, subdivided into five broad classes – receptor tyrosine kinase signaling, MAPK signaling, PI3K/mTOR signaling, cell-cycle control, and DNA damage/apoptotic signaling. We observed numerous differential responses to PI3K targeting in PTEN-proficient versus -deficient lines – typically the PI3K-targeting agents with AZD6244 had no additive impact in PTEN-proficient lines when compared with AZD6244 alone. This suggests that the activation of PI3K signaling may not represent a critical molecular response to MAPK inhibition in PTEN-proficient lines *in vitro* (Fig. 3D). As a counterpoint to these findings, we observed potentiation of AKT phosphorylation at the canonical PDK1 (Thr308) and mTORC2 (Ser473) target sites in AZD6244-treated PTEN-deficient (but not PTEN-proficient) lines, suggesting the activation of PI3K signaling in response to MAPK inhibition in a *PTEN*-dependent manner. Notably, in all PTEN-deficient lines tested, p110β/δ inhibition with AZD8186 was more effective at suppressing AZD6244-mediated AKT phosphorylation at either site than p110α inhibition, suggesting a predominant role for p110β/δ in a PTEN-deficient setting. Our RPPA analyses also showed that inhibition of MEK1/2 and p110β/δ, through coadministration of AZD6244 and AZD8186, impacted several cellular signaling pathways/networks by suppressing key nodes controlling PI3K/mTOR signaling, and cell-cycle progression. Amongst these, suppression of downstream targets/effectors of mTORC1, such as phosphorylation of 4EBP1 (Thr37/46 and Ser65) or RPS6 (Ser235/236 and Ser240/244), and suppression of key cell-cycle control nodes, such as expression of c-MYC and survivin or phosphorylation of cyclin D1 (Thr286) and Rb (Ser807/811), suggested that p110β/δ inhibition may counteract AKT/mTORC1-dependent cell-cycle progression potentiated by MAPK inhibition (Fig. 3D).

In addition to the suppression of PI3K/mTORC1 signaling, RPPA analysis indicated decreased cell-cycle progression (decreased phospho-Rb) following combined inhibition of MEK1/2 and p110β/δ selectively in PTEN-deficient lines (Fig. 3D). This was validated by immunoblotting, with the combination of AZD6244/AZD8186 resulting in ablation of Rb (Ser795) phosphorylation and downregulation of cyclin D1 (Supplementary Fig. S7G and S7H). Intriguingly, AZD6244-mediated inhibition of MAPK signaling suppressed Rb phosphorylation and cyclin D1 expression in PTEN-proficient A375 cells, but AZD8186 had no additive effect (Supplementary Fig. S7G and S7H). Irrespective of *PTEN* status, MAPK inhibition attenuated Rb phosphorylation and cyclin D1 expression; notably, however, in PTEN-deficient WM266.4 cells, p110β/δ inhibition had an additive effect (Supplementary Fig. S7G and S7H). This suggests that MAPK and PI3Kβ signaling converge upon Rb via different mechanisms, such as transcriptional control of *CCND1* (encoding cyclin D1) by ERK1/2 and/or MYC, downstream of MAPK signaling, versus proteolytic degradation of cyclin D1 by GSK3β (48), the kinase activity that is in turn inhibited by PI3K/AKT/mTOR signaling (49).

**Figure 4.**
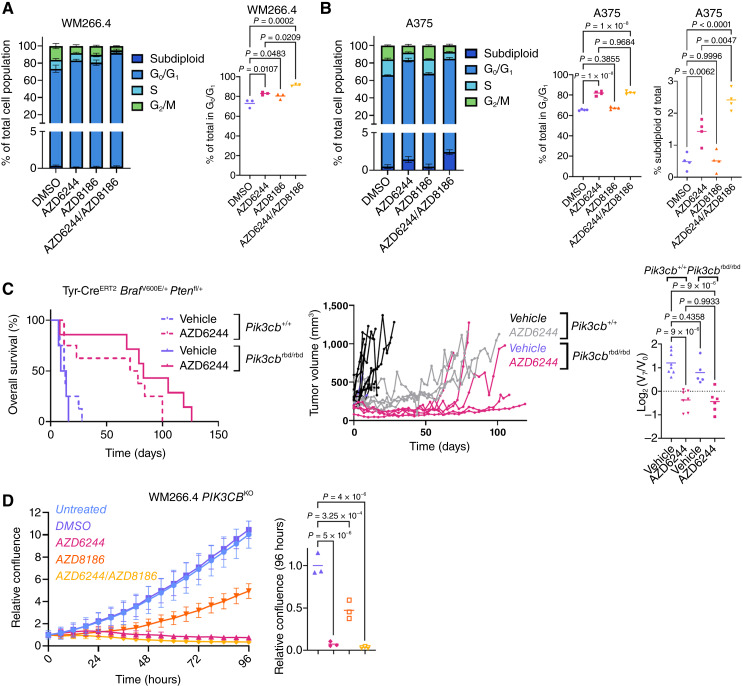
Genetic targeting of p110β and its interaction with RAC1 sensitizes to MEK1/2 inhibition *in vitro* and *in vivo.***A,** Left, flow cytometry–based cell-cycle profiling of WM266.4 cells following indicated 24-hour treatment. Right, proportion of WM266.4 cells in G_1_/S at 24 hours. *n* = 3 independent experiments. *P* values calculated by one-way ANOVA corrected for multiple comparisons (Tukey). **B,** Left, flow cytometry–based cell-cycle profiling of A375 cells following indicated 24 hours treatment. Middle, proportion of A375 cells in G_1_/S at 24 hours. Right, proportion of subdiploid A375 cells at 24 hours. *n* = 4 independent experiments. *P* values calculated by one-way ANOVA corrected for multiple comparisons (Tukey). **C,** Left, Kaplan–Meier overall survival of BRAF PTEN mice treated with vehicle or AZD6244. BRAF PTEN + vehicle (*n* = 8; median survival, 12.5 days) vs. BRAF PTEN + AZD6244 (*n* = 8; median survival, 74.5 days), *P* = 0.0096; BRAF PTEN PIK3CB-mut + vehicle (*n* = 4; median survival, 11.5 days) vs. BRAF PTEN PIK3CB-mut + AZD6244 (*n* = 7; median survival, 83 days), *P* = 0.0082; BRAF PTEN + AZD6244 (*n* = 8; median survival, 74.5 days) vs. BRAF PTEN PIK3CB-mut + AZD6244 (*n* = 7; median survival, 83 days), *P* = 0.1643. *P* values calculated using the log-rank (Mantel–Cox) test. Middle, longitudinal growth of individual tumors from vehicle-treated (*n* = 8) vs. AZD6244-treated (*n* = 7) BRAF PTEN cohorts and vehicle-treated (*n* = 5) vs. AZD6244-treated (*n* = 7) BRAF PTEN PIK3CB-mut cohorts. Right, relative change in tumor volume over the first 7 days of indicated treatment. BRAF PTEN + vehicle (*n* = 8), BRAF PTEN + AZD6244 (*n* = 7), BRAF PTEN PIK3CB-mut + vehicle (*n* = 5), BRAF PTEN PIK3CB-mut + AZD6244 (*n* = 6); *P* values calculated by one-way ANOVA corrected for multiple comparisons (Tukey). Center line, mean. Note that the BRAF PTEN treatment datasets are reproduced from Fig. 2A and C. **D,** Left, relative confluence of WM266.4 *PIK3CB*^KO^ cells treated with indicated treatments over the 96-hour period. Representative of a minimum of three independent experiments and three technical replicates. Data, mean ± SEM (confluence relative to starting point). Right, relative change in confluence of WM266.4 *PIK3CB*^KO^ cells over the indicated 96-hour period. Center line, mean. *P* values calculated by one-way ANOVA corrected for multiple comparisons (Tukey).

Given the observed impact on signaling nodes including mTORC1 signaling and Rb phosphorylation, it seemed likely that MAPK and p110β targeting therapies may also impact cell-cycle progression, thus accounting for the observed growth defect *in vitro*. We therefore examined the impact of each MAPK and PI3K/AKT/mTOR mono- or combination therapy on cell-cycle progression in synchronous WM266.4 (Fig. 4A; Supplementary Fig. S8A) or A375 (Fig. 4B; Supplementary Fig. S8B) cultures. MAPK inhibition increased the proportion of cells in G_0_/G_1,_ with a concomitant decrease in the number of cells in the S- and G_2_/M-phases, 24 hours after treatment. Crucially, although AZD8186 had no further impact on any A375 subpopulation, it substantially increased the proportion of WM266.4 cells in G_0_/G_1_, compared with MAPK inhibition alone (Fig. 4A and B). Notably, MAPK inhibition was sufficient to drive a small, yet significant increase in the subdiploid, apoptotic population in the A375 line, which was in turn enhanced by combination with AZD8186 (Fig. 4B, right).

The impact upon cell-cycle progression of cotargeting MAPK (AZD6244) and p110β/δ (AZD8186) in WM266.4 was phenocopied by cotargeting of downstream kinases AKT (AZD5363) and mTOR (AZD2014; Supplementary Fig. S8A). Cotargeting of MAPK with p110α/δ (AZD8835) in this line resulted in no additional benefit beyond that observed with monotherapy AZD6244 (Supplementary Fig. S8A). In contrast to WM266.4, combination treatment had no additional impact on the AZD6244-induced cell-cycle arrest of PTEN-proficient A375 cells (Supplementary Fig. S8B). Notably, a similar pattern was observed in the PTEN-deficient WM793 line, which seemed resistant to cell-cycle arrest and apoptosis induction upon PI3K/AKT/mTOR targeting (Supplementary Fig. S8C and S8D), consistent with the lack of impact observed via confluence measurements (Supplementary Fig. S6C).

### Genetic targeting of the p110β–RAC1 interaction phenocopies ablation of *Prex2 in vivo* and *in vitro*

Targeting RAC1 activity via genetic disruption of PREX2 can enhance sensitivity to MAPK inhibition in *BRAF*-V600E–driven melanoma *in vivo*, although pharmacologic targeting of the RAC1 effector p110β also seems capable of enhancing responses to MAPK inhibition *in vitro*. This correlation suggests that RAC1-mediated p110β activation may play a role in response or resistance to targeted MAPK inhibition. The interaction between RAC1 and p110β, which stimulates p110β catalytic activity, is mediated by the canonical RAS-binding domain (RBD) of p110β. Targeting this interaction may therefore sensitize BRAF-mutant melanoma to MAPK inhibition. We therefore incorporated a mutant *Pik3cb*^S205D,K224A^ allele (*Pik3cb*^rbd^), encoding p110β with a nonfunctional RBD knocked-in to the endogenous *Pik3cb* locus (12), in the BRAF PTEN line, generating Tyr*-*Cre^ERT2^*Braf*^V600E/+^*Pten*^fl/+^*Pik3cb*^rbd/rbd^ mice (BRAF PTEN PIK3CBmut; Fig. 4C; Supplementary Fig. S8E). This *Pik3cb* mutant had little-to-no impact upon disease trajectory, with naevus onset and melanoma initiation similar to BRAF PTEN controls, although *Pik3cb* mutation did seem to result in improved overall survival (Supplementary Fig. S8E). In line with the prediction that disrupting the RAC1–p110β interaction might phenocopy deletion or inactivating mutation of *Prex2*, melanomas arising in the BRAF PTEN PIK3CBmut model were acutely sensitive to MAPK inhibition in the short term, and mice exhibited prolonged overall survival and a delay in the onset of therapeutic resistance compared with BRAF PTEN controls (Fig. 4C; Supplementary Fig. S8E).

We then turned to CRISPR/Cas9-mediated genome editing to assess the impact of p110β loss in the WM266.4 melanoma line *in vitro*. In this case, TIDE analysis indicated that 73.2% of *PIK3CB* transcripts were generated from disrupted genomic sequences (Supplementary Fig. S8F). In line with *vivo* data, this analysis demonstrated that *PIK3CB* expression was dispensable for growth of WM266.4 cells, but that disruption induced sensitivity to AZD6244 (Fig. 4D). Crucially, *PIK3CB* disruption also abrogated the impact of AZD8186 when in combination with AZD6244, suggesting that, the additive antiproliferative impact of AZD8186 here is mediated by the inhibition of p110β rather than p110δ (Fig. 4D). Additionally, AZD8186 monotherapy partially suppressed growth in this p110β-deficient setting, implying that p110δ may function in growth of WM266.4 under basal conditions, or in response to loss of p110β (Fig. 4D).

### Cotargeting of the MAPK and RAC1/p110β pathway has therapeutic efficacy in human PTEN-deficient melanoma xenografts *in vivo*

We next tested whether the additive impact of pharmacologic inhibition of p110β/δ with AZD8186 upon MAPK targeting could be recapitulated *in vivo*. To do so, WM266.4 or A375 cells were engrafted subcutaneously into the flank of athymic CD1-Foxn1^nu^ mice. Mice were enrolled into appropriate treatment groups once engrafted tumors had reached a diameter ≥7 mm, and therapeutic response was measured both in terms of primary tumor growth/regression and overall survival.

**Figure 5.**
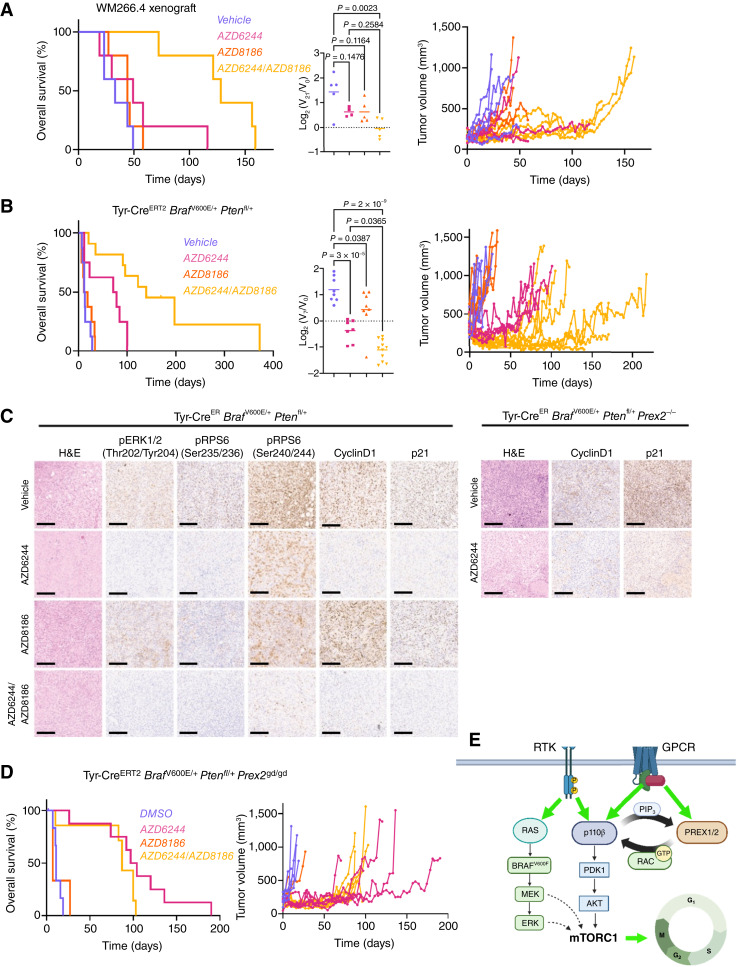
Therapeutic targeting of MEK1/2 and the RAC1 effector p110β has marked therapeutic efficacy in melanoma *in vivo*. **A,** Left, Kaplan–Meier overall survival of mice harboring WM266.4 subcutaneous xenografts treated with vehicle (*n* = 5; median survival, 33 days), AZD6244 (*n* = 5; median survival, 49 days), AZD8186 (*n* = 5; median survival, 44 days), or AZD6244/AZD8186 (*n* = 5; median survival, 128 days). *P* values calculated using the log-rank (Mantel–Cox) test: vehicle vs. AZD6244, *P* = 0.2269; vehicle vs. AZD8186, *P* = 0.3091; vehicle vs. AZD6244/AZD8186, *P* = 0.0017 and AZD6244 vs. AZD6244/AZD8186, *P* = 0.0064. Middle, relative change in tumor volume of WM266.4 xenografts over the first 21 days of indicated treatment. Vehicle (*n* = 5), AZD6244 (*n* = 4), AZD8186 (*n* = 5), AZD6244/AZD8186 (*n* = 5). *P* values calculated by one-way ANOVA corrected for multiple comparisons (Tukey). Right, longitudinal growth of individual WM266.4 xenografts treated with vehicle (*n* = 5) vs. AZD6244 (*n* = 5), AZD8186 (*n* = 5), or AZD6244/AZD8186 (*n* = 5). **B,** Left, Kaplan–Meier overall survival of BRAF PTEN mice treated with vehicle (*n* = 8; median survival, 12.5 days), AZD6244 (*n* = 8; median survival, 74.5 days), AZD8186 (*n* = 8; median survival, 15 days), or AZD6244/AZD8186 (*n* = 8; median survival, 139 days). *P* values calculated using the log-rank (Mantel–Cox) test: vehicle vs. AZD6244, *P* = 0.009550; vehicle vs. AZD8186, *P* = 0.437704; vehicle vs. AZD6244/AZD8186, *P* = 0.000012 and AZD6244 vs. AZD6244/AZD8186, *P* = 0.004813. Middle, relative change in tumor volume of BRAF PTEN mice over the first 7 days of indicated treatment. Vehicle (*n* = 8), AZD6244 (*n* = 7), AZD8186 (*n* = 8), AZD6244/AZD8186 (*n* = 11). *P* values calculated by one-way ANOVA corrected for multiple comparisons (Tukey). Right, longitudinal growth of individual tumors from vehicle-treated (*n* = 8) vs. AZD6244-treated (*n* = 7), AZD8186-treated (*n* = 8), and AZD6244/AZD8186-treated (*n* = 11) BRAF PTEN cohorts. Note that the BRAF PTEN vehicle- and AZD6244-treated cohort data are also used in Figs. 2 and 3. **C,** Left, representative H&E staining and IHC against pERK1/2 (Thr202/Tyr204), pRPS6 (Ser235/Ser236), pRPS6 (Ser240/Ser244), cyclin D1, and p21 in BRAF PTEN mice treated with indicated treatment. Right, representative H&E staining and IHC against cyclin D1 and p21 in tumor sections from BRAF PTEN PREX2 mice treated with vehicle or AZD6244. **D,** Top, Kaplan–Meier overall survival of BRAF PTEN PREX2-GD mice treated with vehicle (*n* = 8; median survival, 11 days), AZD6244 (*n* = 8; median survival, 100.5 days), AZD8186 (*n* = 4; median survival, 7 days), or AZD6244/AZD8186 (*n* = 7; median survival, 87 days). *P* values calculated using the log-rank (Mantel–Cox) test: vehicle vs. AZD6244, *P* = 0.00008; vehicle vs. AZD8186, *P* = 0.73075; vehicle vs. AZD6244/AZD8186, *P* = 0.00328 and AZD6244 vs. AZD6244/AZD8186, *P* = 0.11574. Bottom, longitudinal growth of individual tumors from vehicle-treated (*n* = 7) vs. AZD6244-treated (*n* = 6), AZD8186-treated (*n* = 3), and AZD6244/AZD8186-treated (*n* = 7) BRAF PTEN PREX2-GD cohorts. NB: the BRAF PTEN PREX2-GD vehicle- and AZD6244-treated cohort data are also used in Fig. 2C. **E,** Schematic of proposed proproliferative relationship between p110β and PREX2 in melanoma *in vivo*. GPCR, G-protein–coupled receptor; PIP_3_, phosphatidylinositol-3,4,5-trisphosphate; RTK, receptor tyrosine kinase. [**E,** Created in BioRender. Campbell, A. (2025), https://BioRender.com/a89p130.]

In contrast to our *in vitro* data, MEK1/2 inhibition alone had an immediate impact on tumor growth in both models, slowing the growth of WM266.4 xenografts and driving regression of A375 xenografts over the first 21 and 14 days of treatment, respectively (Fig. 5A; Supplementary Fig. S9A, S9F, and S9G). Nonetheless, combined MEK1/2 and p110β inhibition had no additive effect on tumor volume in A375 derived tumors, although it did drive a modest increase in overall survival (Supplementary Fig. S9A). As predicted by our prior *in vitro* and *in vivo* data, the same inhibitor combination (AZD6244/AZD8186) had a marked benefit, when compared with MEK1/2 inhibition alone in WM266.4 xenografts, driving tumor regression and significantly increasing overall survival (Fig. 5A).

To position these data with respect to current clinical approaches for *BRAF*-mutant melanoma, and in line with earlier *in vitro* experiments, we next compared the impact of administration of AZD8186 or targeted deletion of *PREX2* (via CRISPR/Cas9) upon efficacy of the cobimetinib/dabrafenib doublet MAPK-targeting regimen. This doublet therapy was highly effective, leading to rapid tumor regression and stasis over a prolonged period (>150 days). Triplet combination incorporating pharmacologic targeting of p110β/δ with AZD8186 was well tolerated but had no additional benefit over the span of this experiment. Similarly, genetic targeting of *PREX2* had no impact upon tumor take or growth kinetics under basal conditions and provided no additive benefit to the cobimetinib/dabrafenib doublet (Supplementary Fig. S9B–S9D). In the longer term, a key measure of success for these triplet approaches may be impact upon the emergence of resistance over time.

### Cotargeting of the MAPK and RAC1/p110β pathway has therapeutic efficacy in melanoma GEMMs

Having demonstrated that the significant translational potential of cotargeting of MAPK and PREX2/RAC1/p110β, we next tested whether the same was true in immunocompetent, autochthonous melanoma GEMMs. As previously, BRAF PTEN or BRAF P53 mice, bearing a minimum of one cutaneous melanoma (diameter >7 mm), were continuously treated to a defined endpoint (tumor size ≥15 mm, or ulceration). Despite a slowing of tumor growth over the first 7 days of treatment, AZD8186 monotherapy did not positively impact overall survival, nor did it prolong response in either the BRAF PTEN or BRAF P53 models (Fig. 5B; Supplementary Fig. S9E, S9H, and S9I). Compared with AZD6244 alone, AZD6244/AZD8186 had no additional impact on tumor regression in BRAF P53 mice, but it did drive a significant increase in overall survival and longevity of response relative to AZD6244 monotherapy (Supplementary Fig. S9E). As predicted, the combined treatment of BRAF PTEN mice with AZD6244/AZD8186 was highly effective, resulting in attenuated tumor growth, tumor regression over the first 7 days of treatment, and extended overall survival (Fig. 5B). Comparison to doublet MAPK targeting in this model suggests inferiority (Supplementary Fig. S4A), but the potential for use as an agent targeting acquired clinical resistance to doublet approaches should be explored.

To understand whether the mechanisms associated with delayed onset of resistance in the BRAF PTEN model of melanoma aligned to those identified *in vitro*, we analyzed the expression patterns of key biomarkers in BRAF PTEN melanomas *in vivo*. both AZD6244 and AZD6244/AZD8186 combination treatment equally suppressed MAPK signaling (pERK Thr202/Tyr204), RPS6 (Ser235/236) phosphorylation, and the expression and nuclear accumulation of cyclin D1 and p21, only RPS6 (Ser240/244) phosphorylation seemed more significantly suppressed by the combination treatment than AZD6244 monotherapy, at this timepoint (Fig. 5C). Moreover, the expression and nuclear accumulation of cyclin D1 and p21 were also suppressed in AZD6244-treated BRAF PTEN PREX2 melanomas (Fig. 5C, right). In support of this impact upon proproliferative signaling, inhibited growth *in vivo* is recapitulated by transcriptional profiling of BRAF PTEN tumors in response to short-term treatment. As would be expected in a MAPK-driven tumor model, MEK1/2 inhibition with AZD6244 resulted in dramatic remodeling of the transcriptional landscape, which was contrasted with the minimal impact of p110α (AZD8835) or p110β/δ (AZD8186) monotherapy (Supplementary Fig. S10A). Despite the lack of efficacy as monotherapy, both AZD8186 and AZD8835 amplified the transcriptional impact of AZD6244 (Supplementary Fig. S10A). Moreover, this specifically translates into an impact upon proproliferative gene signatures, with suppression of transcripts associated with activation of both E2F and cMyc transcriptional programs following MEK1/2 inhibition, and deeper suppression of these same targets upon combined inhibition of MEK1/2 and p110β/δ (Supplementary Fig. S10B).

Finally, to consolidate the link between genetic targeting of PREX2 and pharmacologic inhibition of p110β/δ with AZD8186, we again used BRAF PTEN PREX2-GD. Our hypothesis was that if the enhanced response to MAPK observed following genetic targeting of PREX2 or pharmacologic targeting of p110β/δ acted via the same mechanism, no additional benefit for should be elicited by targeting melanomas lacking functional PREX2 with AZD8186. As predicted, AZD8186 monotherapy had no impact upon tumor growth or overall survival, although critically, the combination of AZD6244 and AZD8186 in this setting provided no additional benefit when compared with AZD6244 monotherapy (Fig. 5D). This suggests an epistatic relationship between PREX2 and p110β/δ and substantiates the link between the two.

## Discussion

We have used genetic deletion and loss-of-GEF-function mutation to model the effects of systemic inhibition of PREX2 signaling. We find that PREX2 loss or mutation strongly cooperates with MEK1/2 inhibition, in a manner phenocopied by, and epistatic to, inhibition p110β/δ, to suppress growth of complex melanoma models, in a genotype-specific manner both *in vitro* and *in vivo*. Consistent with our findings, several studies have demonstrated that multiple PTEN-deficient tumor types are reliant on p110β activity, with p110β–selective inhibition or genetic deletion of *PIK3CB*—but not *PIK3CA (*p110α)—sufficient to perturb PI3K/AKT signaling and abrogate tumor cell growth *in vitro* and *in vivo*, with combination therapies showing the highest potency (33, 47, 50, 51).

The observed lack of monotherapy impact of p110β inhibition is supported by other studies, indicating that *BRAF*V600E *PTEN* melanoma is refractory to p110β monotherapy blockade, with combined inhibition of p110α, p110γ, and p110δ required to arrest its proliferation. Moreover, *RAC1-*P29S–mutant melanoma cells have been recently shown to be refractory to p110β selective monotherapy, albeit *in vitro*, even though RAC1 activation directly regulates p110β (12, 52). In line with our data, these studies imply that combination therapeutic approaches may be the most effective way to deploy p110β inhibitors clinically. Our data suggest that p110β inhibition selectively sensitizes cells to MEK inhibition in the subset of PTEN-deficient melanomas harboring hyperactivation of RAC1 signaling, or broader dysregulation of the PREX2/RAC1/PI3Kβ axis. Nevertheless, our data do not preclude the activity of the other type I PI3K isoforms in melanoma; indeed, we present data that hint at activity of other isoforms in our models. Moreover, these data, along with those of other groups, suggest that combination therapy with isoform-specific inhibitors of PI3K, tailored to the patient’s profile, may offer a means to prolong the duration of the response to MAPK-targeted therapies in patients with *BRAF*-mutant melanoma.

Despite almost unparalleled success in the use of targeted therapeutics for patient benefit in melanoma over the last two decades, and extensive research into the mechanisms underlying intrinsic and acquired therapy resistance, refractory or recurrent disease portends a dismal prognosis and remains a pressing problem in the clinic. Here, we demonstrate that genetic or therapeutic targeting of the PREX2/RAC1/p110β pathway can substantially enhance responses to MAPK targeting in *BRAF*-mutant melanoma both *in vitro* and *in vivo*, presumably through suppression of AKT/mTOR driven cell-cycle progression (Fig. 5E). Given that clinically relevant p110β inhibitors are both currently available and well-tolerated, our research suggests a therapeutic approach that could be of significant benefit to patients in the future.

## Supplementary Material

Supplementary Figures S1-S10 Supplementary Figures S1-S10 with associated figure legends.

Supplementary Table S1 Reverse Phase Protein Array antibodies

Supplementary Table S2 Cancer subtypes abbreviations
