# Supplementary material for: Targeting the PREX2/RAC1/PI3Kβ Signaling Axis Confers Sensitivity to Clinically Relevant Therapeutic Approaches in Melanoma
Source: Cancer Res. 2024 Dec 5;85(4):808–24. doi: 10.1158/0008-5472.CAN-23-2814 (PMC11831108; doi:10.1158/0008-5472.CAN-23-2814)
Supplement: Supplementary Figures S1-S10 — with associated figure legends. [file can-23-2814_supplementary_figures_s1-s10_suppsf1-10.pdf]

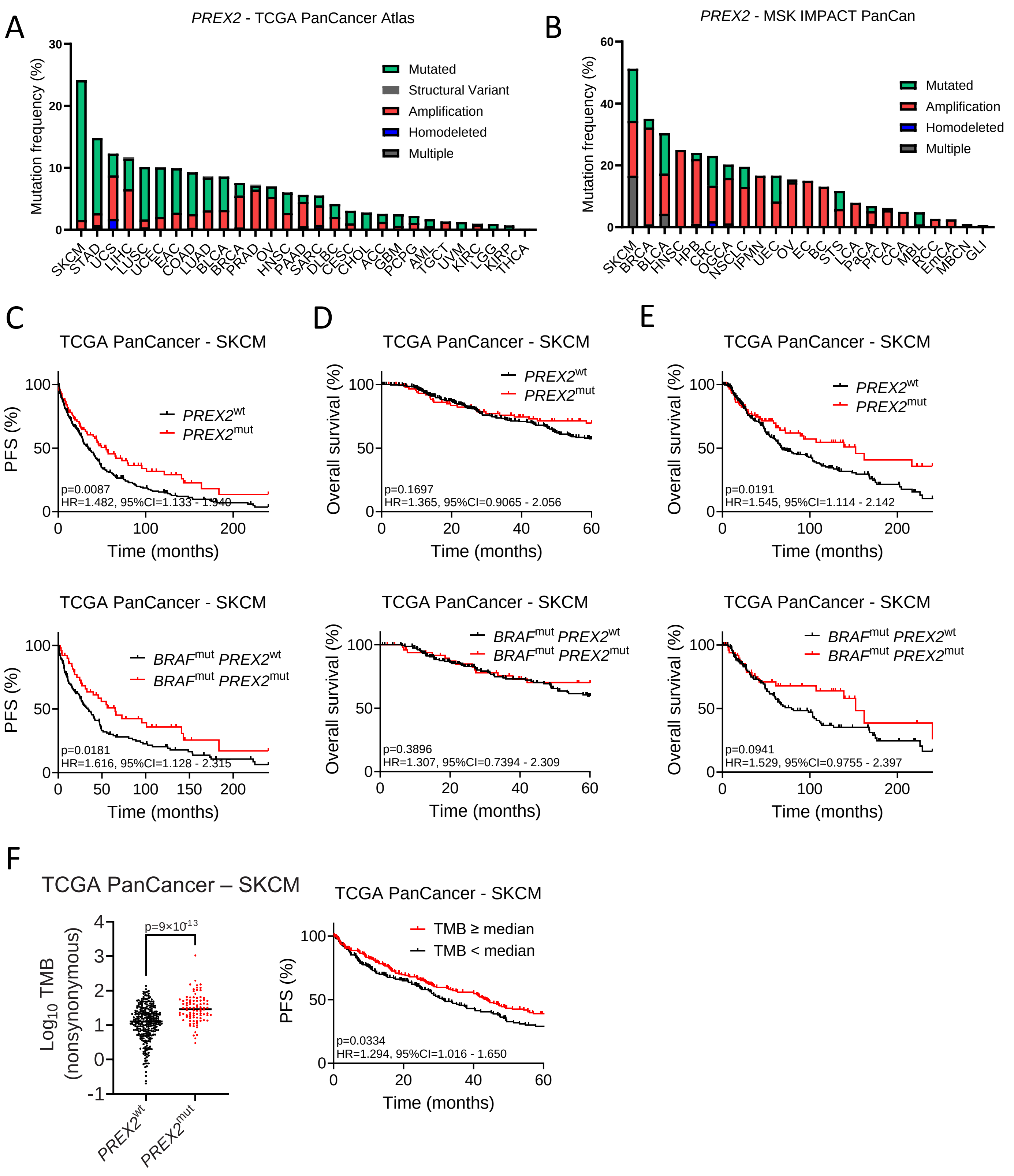

SUPPLEMENTARY 1

Supplementary Fig. S1: The impact of *PREX2* mutation in human cancers. A) *PREX2* mutation frequency and type across indicated cancers in/from the TCGA PanCancer Atlas patient cohort. B) *PREX2* mutation frequency and type across indicated cancer types in the MSK-IMPACT PanCancer patient cohort. Please see Supplementary Table S1 for abbreviations. For abbreviations see Table S2. C) Progression-free survival (PFS) of *PREX2* mutant *vs* wild-type cases in the curated cohort of SKCM patients from the TCGA PanCancer cohort, censored at 20 years. Top panel, all cases (n=381; *PREX2* wild-type = 290, *PREX2* mutant = 91); Bottom panel, *BRAF*-mutant cases (n=207; *PREX2* wild-type = 155, *PREX2* mutant = 52). D) Overall survival of *PREX2*-mutant *vs* wild-type SKCM cases from the TCGA PanCancer Atlas curated patient cohort, censored at 5 years. Top panel, all cases (n = 380; *PREX2* wild-type = 289, *PREX2* mutant = 91); Bottom panel, *BRAF*-mutant cases (n = 206; *PREX2* wild-type = 154, *PREX2* mutant = 52). E) Overall survival of *PREX2*-mutant *vs* wild-type SKCM cases from the TCGA PanCancer Atlas curated patient cohort, censored at 20 years. Top panel, all cases (n = 380; *PREX2* wild-type = 289, *PREX2* mutant = 91); Bottom panel, *BRAF*-mutant cases (n = 206; *PREX2* wild-type = 154, *PREX2* mutant = 52). F) Left panel, total mutation burden (TMB) of *PREX2*-mutant (n = 91) *vs* wild-type (n = 290) SKCM cases from the TCGA PanCancer Atlas curated patient cohort. TMB was calculated by the number of non-synonymous mutations, comprising single nucleotide variants, splice-site variants, and short insertions and deletions (InDels), per Mb of coding regions. Centre line, median TMB. Right panel, progression-free survival (PFS) of SKCM patients stratified by median TMB, censored at 5 years. TMB<median, n = 190, TMB≥median, n = 191.

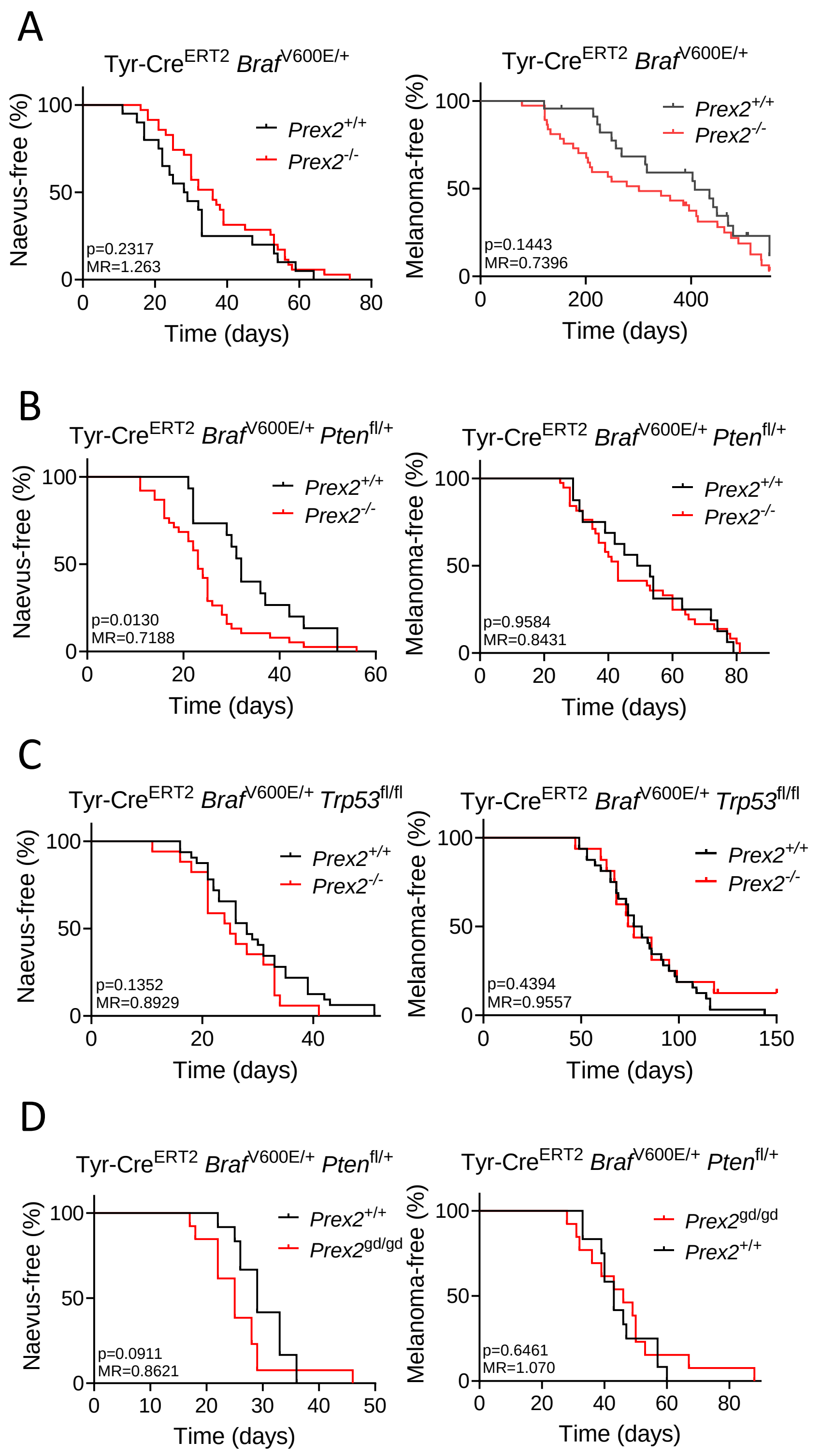

SUPPLEMENTARY 2

Supplementary Fig. S2: Characterisation of the disease trajectory of PREX2-deficient melanoma models. A) Naevus-free (left) and melanoma-free (right) survival of BRAF and BRAF PREX2 cohorts. Tick marks indicate melanoma-free mice censored at indicated times post induction. B) Naevus-free (left) and melanoma-free (right) survival of BRAF PTEN and BRAF PTEN PREX2 cohorts. C) Naevus-free (left) and melanoma-free (right) survival of the indicated BRAF P53 and BRAF P53 PREX2 cohorts. D) Naevus-free (left) and melanoma-free (right) survival of BRAF PTEN and BRAF PTEN PREX2-GD cohorts. MR, median ratio.

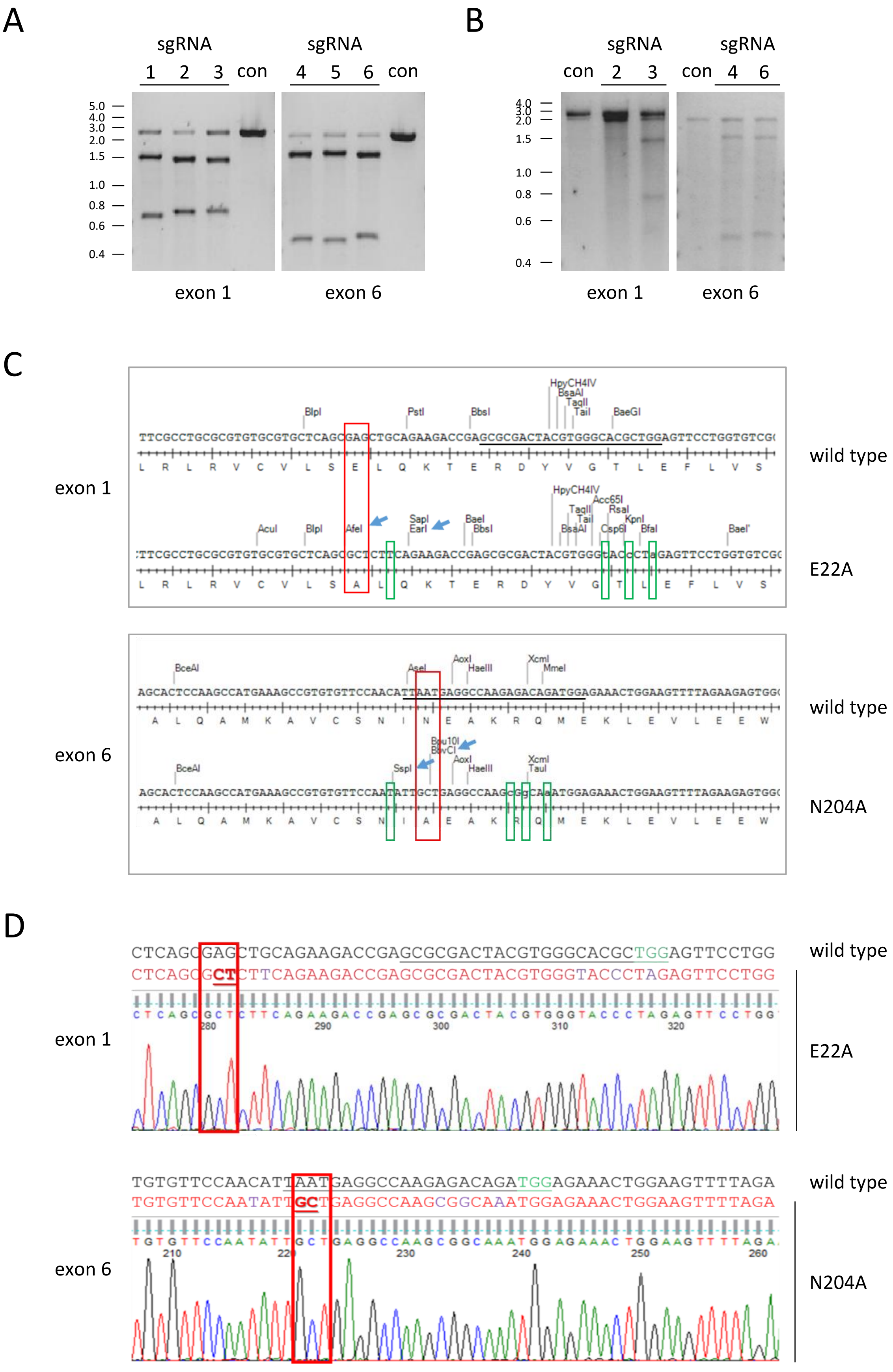

SUPPLEMENTARY 3

Supplementary Fig. S3: Targeting strategy for catalytically inactive *Prex2* transgenic allele. (A) Assessment sgRNA efficiencies *in vitro*. PCR products of *Prex2* mouse genomic sequence encompassing the target sites in exon 1 and exon 6 were cleaved by recombinant Cas9 nuclease in the presence of candidate sgRNAs, as indicated. Control DNA fragments were mock-treated in absence of sgRNA. (B) Assessment sgRNA efficiency in cells. NIH/3T3 cells were transfected with sgRNAs and Cas9 to target sites in exon 1 and exon 6 of *Prex2*. The relevant regions of genomic DNA were amplified, annealed with wild type DNA, and treated with Surveyor nuclease to cut at the mismatches. Wild type DNA fragments were used as controls. (C) Restriction maps of part of the DNA repair templates used to introduce point mutations in *Prex2* exons 1 and 6, compared to the wild type sequence. Red boxes show the nucleotide changes which result in the E22A and N204A mutations. Green boxes highlight silent mutations introduced to create restriction sites or destroy PAM motifs. Blue arrows show restriction enzyme sites useful for screening. (D) Representative sequencing traces of a homozygous *Prex2*^{gd} (*Prex2*$^{E22A/\ E22A;N204A/N204A}$) mouse (red letters) compared to wild type (black letters). The sgRNA sequences are underlined in black, the PAM sites in green. Red boxes show the nucleotide changes introduced to render the protein catalytically inactive. Purple letters show silent point mutations introduced to create restriction enzyme sites and/or destroy PAM motifs.

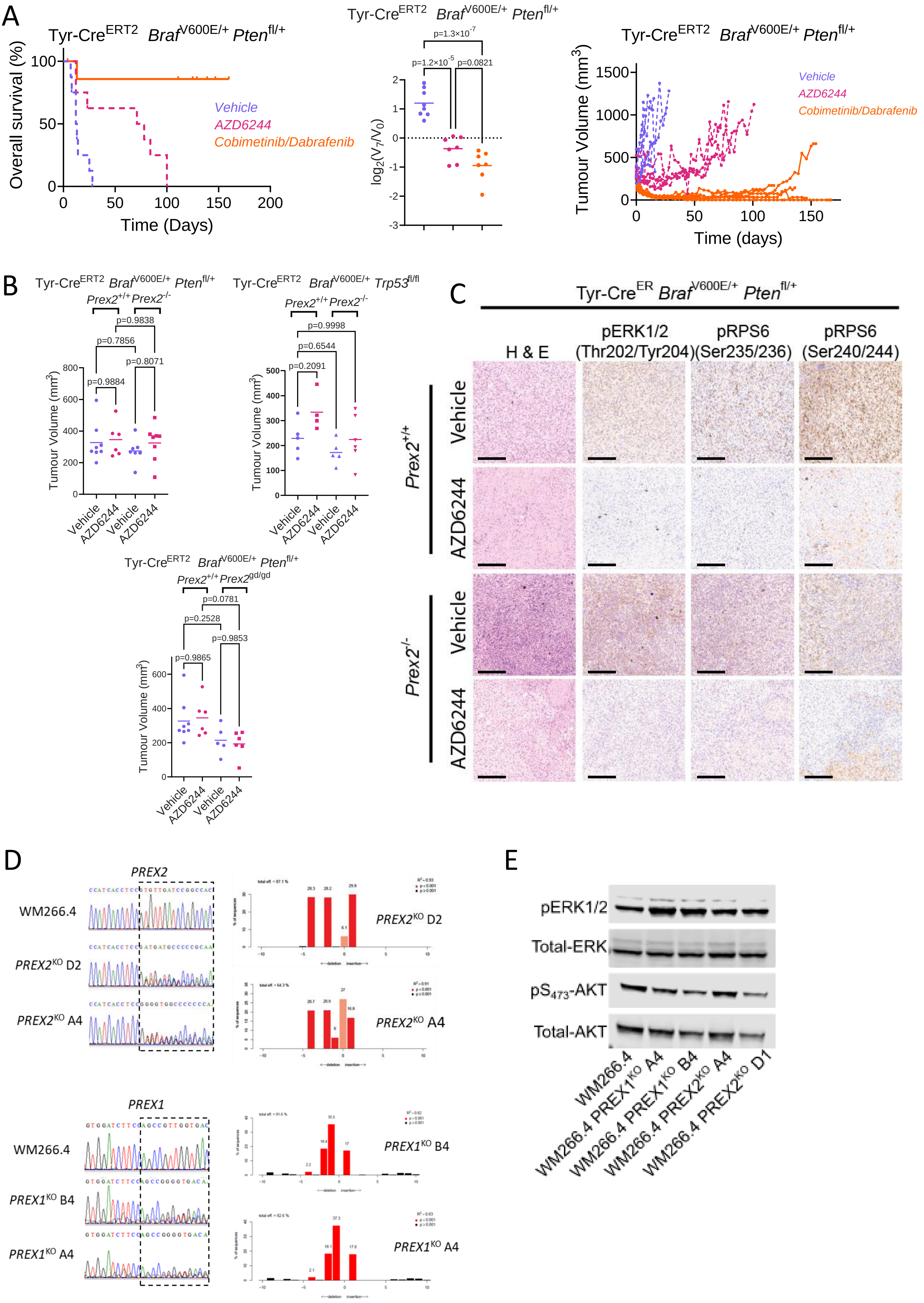

SUPPLEMENTARY 4

Supplementary Fig. S4: Characterising the therapeutic impact of MAPK inhibition in PREX2-deficient melanoma *in vivo* and *in vitro*. A) Left panel, Kaplan–Meier overall survival of BRAF PTEN mice treated with vehicle (n=8; median survival, 12.5 days), AZD6244 (n=8; median survival, 74.5 days), or cobimetinib/dabrafenib (n=8; median survival, undefined). p-values calculated by log-rank (Mantel–Cox) test – vehicle vs AZD6244, p=0.00955; vehicle vs cobimetinib/dabrafenib, p=0.00088 and AZD6244 vs cobimetinib/dabrafenib, p= 0.00241. Centre panel, relative change in tumour volume of BRAF PTEN mice over the first 7 days of indicated treatment. Vehicle (n=8), AZD6244 (n=7) and cobimetinib/dabrafenib (n=7); p-values calculated by one-way ANOVA corrected for multiple comparisons (Tukey). Right panel, longitudinal growth of individual tumours from vehicle-treated (n=8) *vs* AZD6244-treated (n=7) and cobimetinib/dabrafenib-treated (n=8) BRAF PTEN cohorts. Note that the BRAF PTEN vehicle- and AZD6244-treated cohort data are also used in Figs. 2 and 4. B) Pre-treatment tumour volume in cohorts of BRAF PTEN (vehicle, n=8; AZD6244, n=6) *vs* BRAF PTEN PREX2 (vehicle, n=7; AZD6244, n=8) (B), upper left panel; BRAF P53 (vehicle, n=5; AZD6244, n=4) *vs* BRAF P53 PREX2 (vehicle, n=5; AZD6244, n=6), upper right panel; and BRAF PTEN (vehicle, n=8; AZD6244, n=6) *vs* BRAF PTEN PREX2-GD (vehicle, n=5; AZD6244, n=6), lower panel. Note that the same BRAF PTEN treatment datasets are represented in upper right and lower panels. p-values calculated by one-way ANOVA corrected for multiple comparisons (Tukey). C) Representative H&E staining and IHC against phospho-ERK1/2 (Thr202/Tyr204), phospho-RPS6 (Ser235/236), and phospho-RPS6 (Ser240/244) in tumours from BRAF PTEN and BRAF PTEN PREX2 cohorts following 5-day treatment with AZD6244 or vehicle. Scale bar – 200 µm. D) Upper left panel, Sanger sequencing trace depicting region immediately upstream and downstream of the targeted protospacer-adjacent motif (PAM) site in *PREX2* in parental WM266.4 parental and *PREX2*^{KO} lines. Upper right panel, frequency and location of indels upstream and downstream of the targeted cut site in *PREX2*^{KO} lines. Lower left panel, Sanger sequencing trace depicting region immediately upstream and downstream of the targeted PAM site in *PREX1* in WM266.4 parental and *PREX1*^{KO} lines. Lower right panel, frequency and location of indels upstream and downstream of the targeted cut site in *PREX1*^{KO} lines. E) Immunoblotting for the indicated biomarkers in CRISPR/Cas9 edited WM266.4 lines.

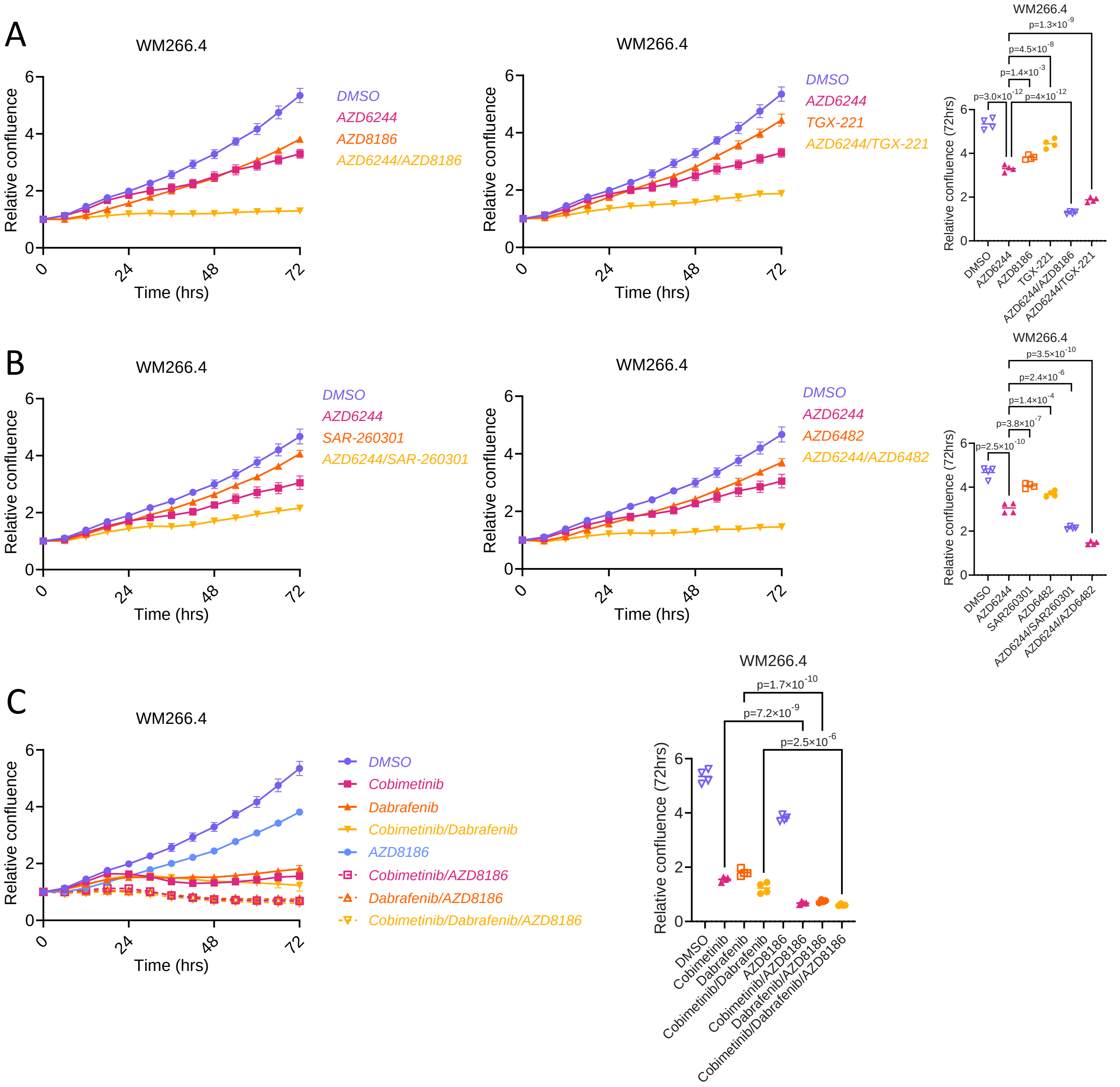

SUPPLEMENTARY 5

Supplementary Fig. S5: Investigating isoform selectivity in MAPK/PI3K co-targeting approaches. A, B) Left and centre panels, relative confluence of WM266.4 cells treated with indicated treatments over 72 hour period. Data, mean ± SEM (confluence relative to starting point). Representative of 4 independent experiments and 3 technical replicates. NB the same DMSO and AZD6244 plots are depicted in each panel. Right panel, relative confluence of WM266.4 cells with indicated treatment following 72 h treatment. Centre line, mean. p-values calculated by one-way ANOVA corrected for multiple comparisons (Tukey). C) Left panel, relative confluence of WM266.4 cells treated with indicated treatments over 72 hour period. Data, mean ± SEM (confluence relative to starting point). Representative of 4 independent experiments and 3 technical replicates. Right panel, relative confluence of WM266.4 cells with indicated treatment following 72 h treatment. Centre line, mean. p-values calculated by one-way ANOVA corrected for multiple comparisons (Tukey). NB the same DMSO and AZD8186 plots are depicted in S5A.

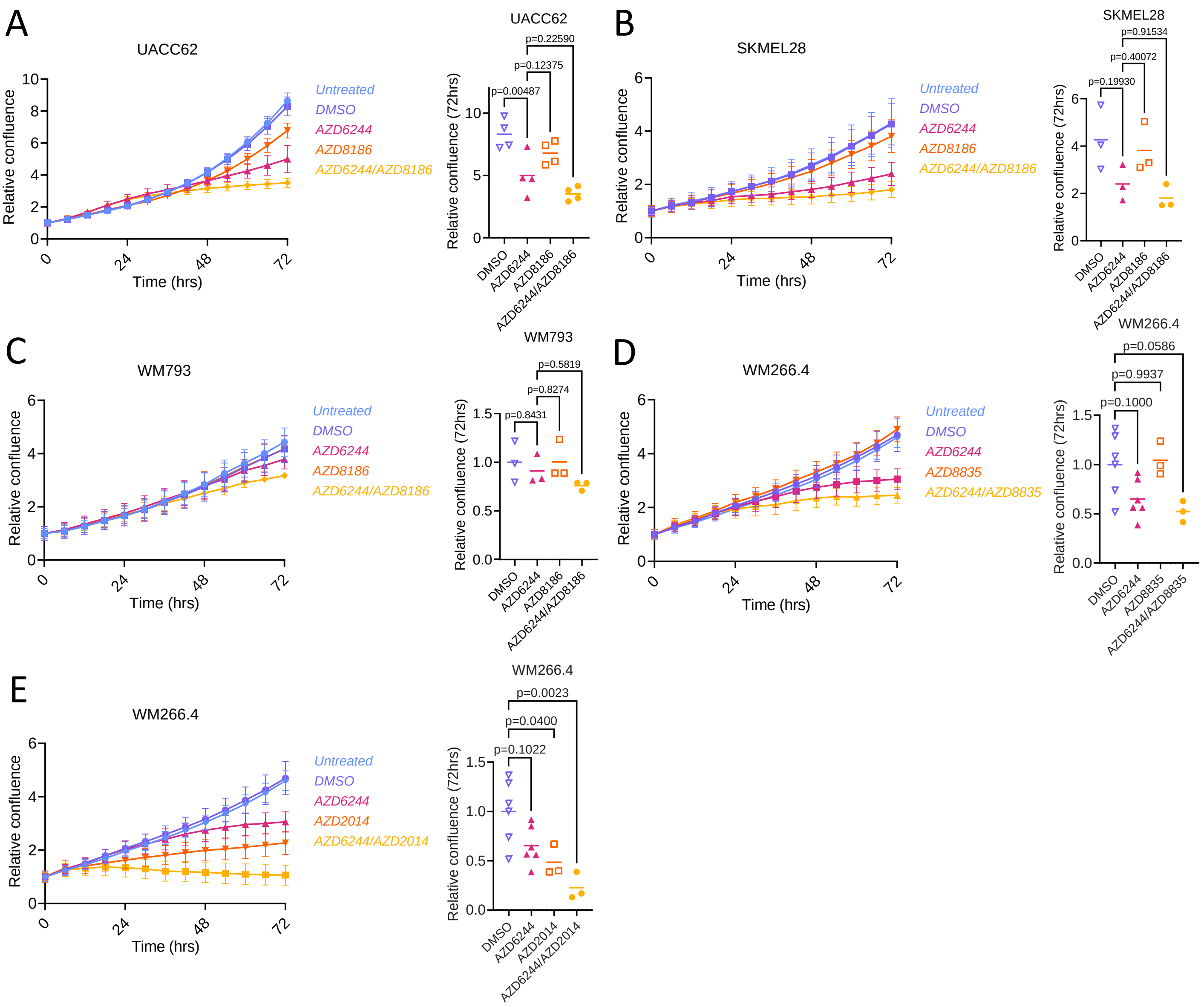

SUPPLEMENTARY 6

Supplementary Fig. S6: Investigating therapeutic efficacy in BRAF PTEN melanoma lines *in vitro*. A) Left panel, relative confluence of UACC62 cells treated with mono- or combination AZD6244, AZD8186 or DMSO over 72 h period. Data, mean ± SEM (confluence relative to starting point). Representative of 4 independent experiments and 3 technical replicates. Right panel, relative confluence of WM266.4 cells with indicated treatment following 72 h treatment. Centre line, mean. p-values calculated by one-way ANOVA corrected for multiple comparisons (Tukey). B) Left panel, relative confluence of SKMEL28 cells treated with mono- or combination AZD6244, AZD8186 or DMSO over 72 h period. Data, mean ± SEM (confluence relative to starting point). Representative of 4 independent experiments and 3 technical replicates. Right panel, relative confluence of WM266.4 cells with indicated treatment following 72 h treatment. Centre line, mean. p-values calculated by one-way ANOVA corrected for multiple comparisons (Tukey). C) Left panel, relative confluence of WM793 cells treated with mono- or combination AZD6244, AZD8186 or DMSO over 72 h period. Data, mean ± SEM (confluence relative to starting point). Representative of 4 independent experiments and 3 technical replicates. Right panel, relative confluence of WM266.4 cells with indicated treatment following 72 h treatment. Centre line, mean. p-values calculated by one-way ANOVA corrected for multiple comparisons (Tukey). D, E) Left panel, relative confluence of WM266.4 cells treated with indicated treatments over 72 h period. Data, mean ± SEM (confluence relative to starting point). Representative of a minimum of 3 independent experiments and 3 technical replicates. Right panel, relative confluence of WM266.4 cells with indicated treatment following 72 h treatment. Centre line, mean. p-values calculated by one-way ANOVA corrected for multiple comparisons (Tukey). NB the same DMSO and AZD6244 plots are depicted in S6D, S6E.

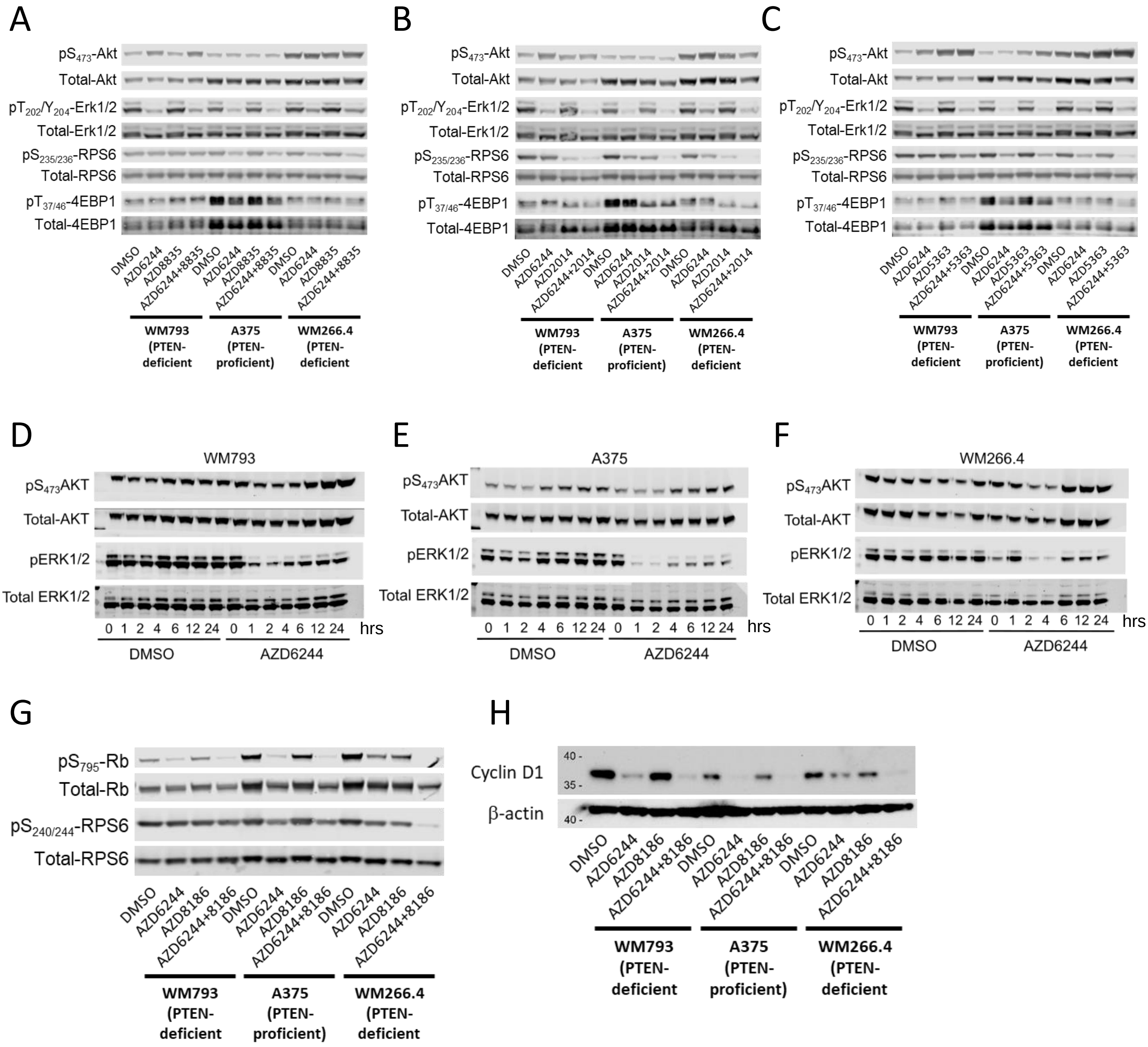

SUPPLEMENTARY 7

Supplementary Fig. S7: Characterisation of biomarker responses in human melanoma derived lines *in vitro*. A-C) Fluorescence labelled (LiCOR) immunoblotting for the indicated activated components of the MAPK–PI3K–mTOR pathway in the indicated human melanoma cells and treated with DMSO or the indicated targeted therapeutics for 24 h. Co-labelled total protein detection for each phospho-protein serves as a sample integrity control. The blots are representative of 3 repeated experiments. D-F) Fluorescence labelled (LiCOR) immunoblotting for ERK1/2 and AKT phosphorylation in WM793, A375 and WM266.4 cells treated with AZD6244 over a 24 h timecourse, with samples collected at the indicated times. Co-labelled total protein detection for each phospho-protein serves as a sample integrity control. G) Fluorescence labelled (LiCOR) immunoblotting for phosphorylated Rb (Ser795) or RPS6 (Ser240/244) in the indicated cell lines following treatment with the indicated targeted therapeutics for 24 h. Co-labelled total protein detection for each phospho-protein serves as a sample integrity control. The blots are representative of 3 repeated experiments. H) Immunoblotting for cyclin D1 expression in the indicated cell lines following treatment with the indicated targeted therapeutics for 24 h. β-actin serves as a sample integrity control. The blots are representative of 3 repeated experiments.

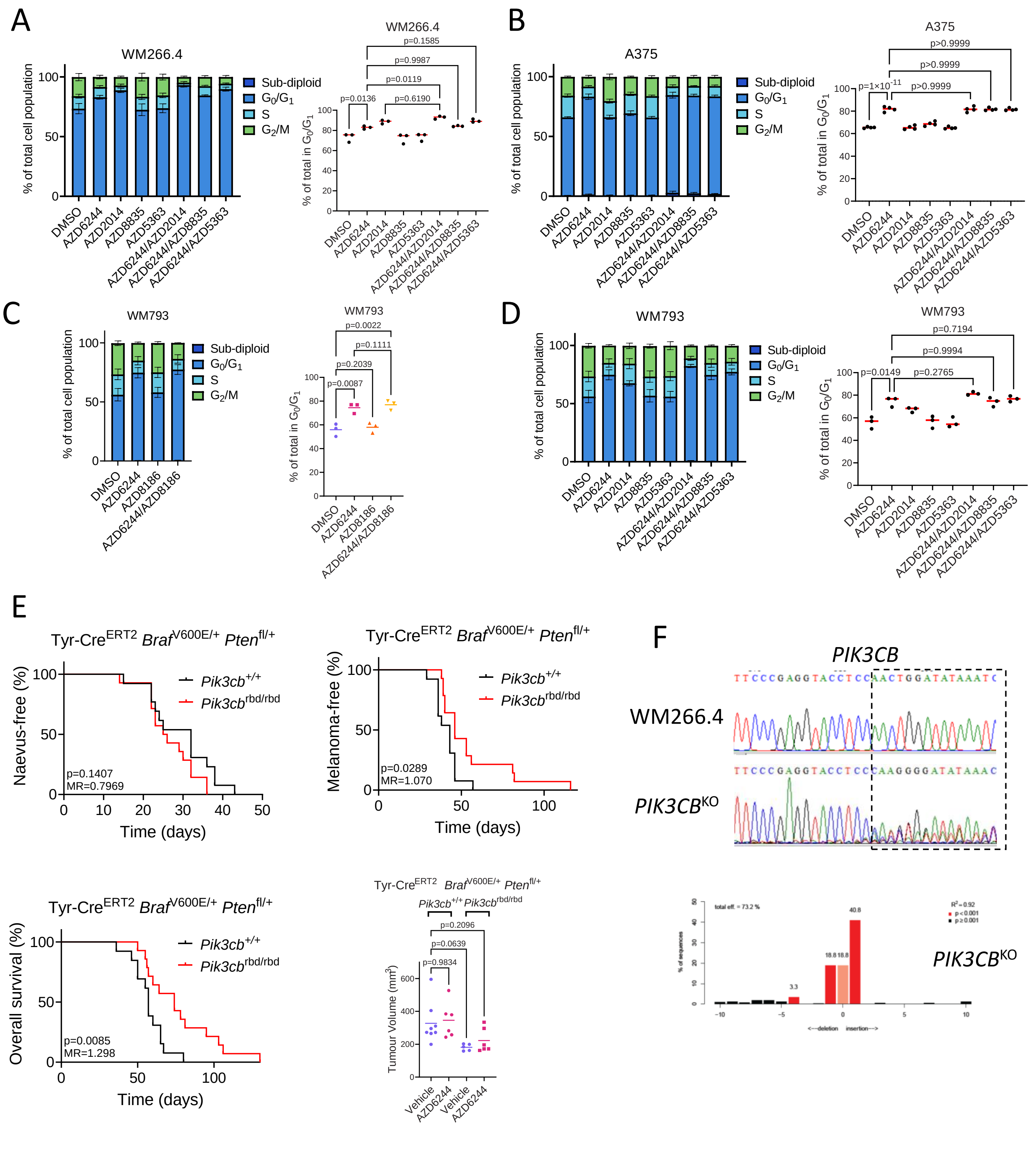

SUPPLEMENTARY 8

Supplementary Fig. S8: Pharamacological and genetic targeting of p110β enhances response to MEK1/2 inhibition *in vivo* and *in vitro*. A) Left panel, flow cytometry–based cell-cycle profiling of WM266.4 cells following indicated 24 h treatment. Right panel, proportion of treated WM266.4 cells in G_1/S at 24 h. n=3 per treatment. Statistical testing by one-way ANOVA corrected for multiple comparisons (Tukey). B) Left panel, flow cytometry–based cell-cycle profiling of A375 cells following indicated 24 h treatment. Right panel, proportion of treated A375 cells in G_1/S at 24 h. n=4 per treatment. Statistical testing by one-way ANOVA corrected for multiple comparisons (Tukey). C,D) Left panels, Flow cytometry–based cell-cycle profiling of WM793 melanoma cells following indicated 24 h treatment. n=3 per treatment. Right panels, proportion of treated WM793 cells in G_1/S at 24 h. Statistical testing by one-way ANOVA corrected for multiple comparisons (Tukey). E) Naevus-free (upper left), melanoma-free (upper right), and overall (lower left) survival of BRAF PTEN *vs* BRAF PTEN PIK3CB cohorts. MR, median ratio. p-values calculated by log-rank (Mantel–Cox) test. Lower right panel, pre-treatment tumour volume in BRAF PTEN *vs* BRAF PTEN PIK3CB cohorts. BRAF PTEN+vehicle, n=8; BRAF PTEN+AZD6244, n=6; BRAF PTEN PIK3CB+vehicle, n=4; BRAF PTEN PIK3CB+AZD6244, n=6. p-values calculated by one-way ANOVA corrected for multiple comparisons (Tukey). NB the BRAF PTEN vehicle- and AZD6244-treated cohort data are also used in S4B. F) Upper panel, Sanger sequencing trace depicting region immediately upstream and downstream of the targeted PAM site in *PIK3CB* in WM266.4 parental and *PIK3CB*^{KO} lines. Lower panel, frequency and location of indels upstream and downstream of the targeted cut site in *PIK3CB*^{KO} line.

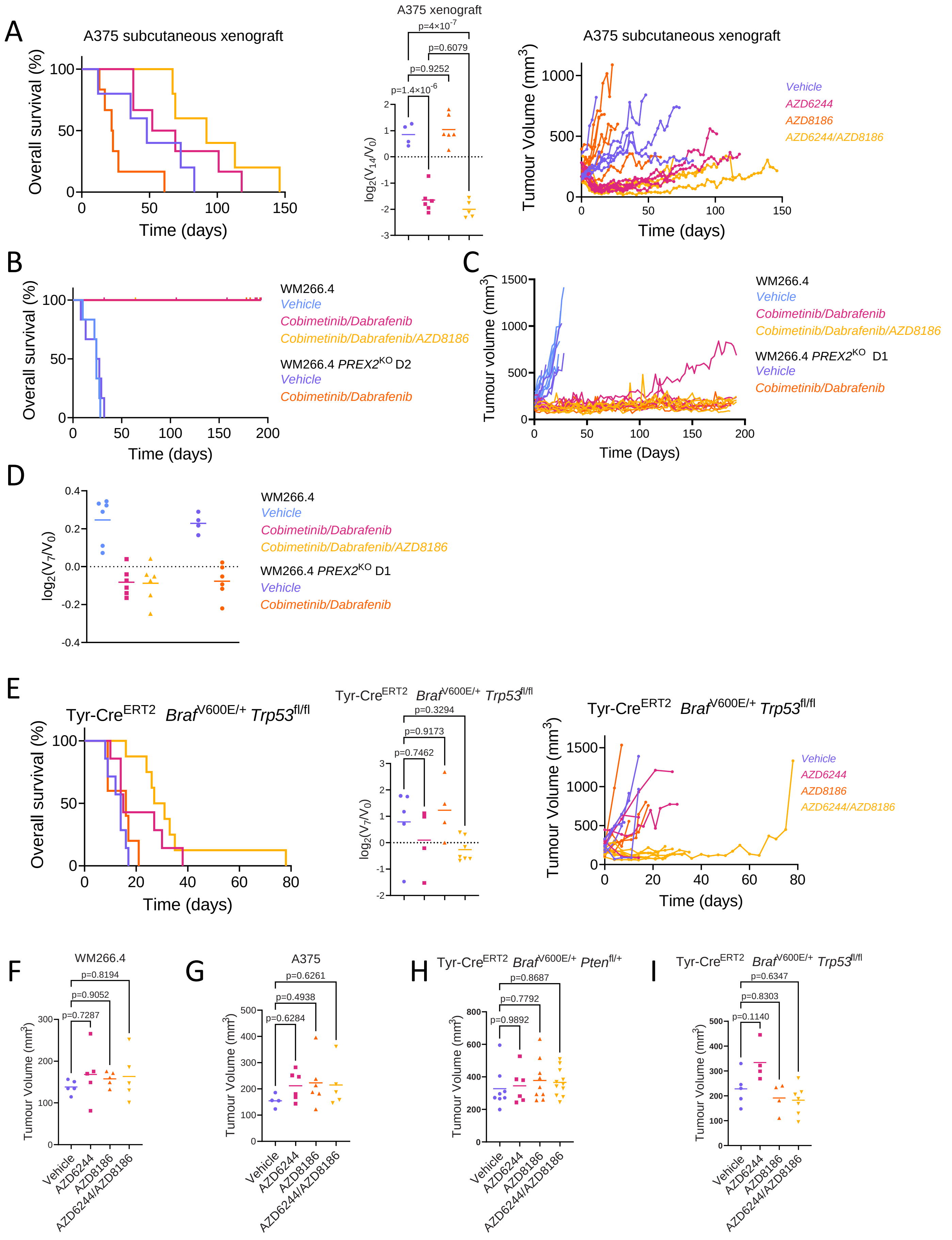

SUPPLEMENTARY 9

Supplementary Fig. S9: Co-targeting of MEK1/2 and p110β has broad therapeutic efficacy in melanoma *in vivo*. A) Left panel, Kaplan–Meier overall survival of mice harbouring A375 subcutaneous xenografts treated with vehicle (n=5; median survival, 48 days), AZD6244 (n=6; median survival, 60.5 days), AZD8186 (n=6; median survival, 22.5 days) or AZD6244/AZD8186 (n=5; median survival, 92 days). p-values calculated by log-rank (Mantel–Cox) test – vehicle vs AZD6244; p=0.3009, vehicle vs AZD8186; p=0.1130, vehicle vs AZD6244/AZD8186; p=0.0554 and AZD6244 vs AZD6244/AZD8186; p=0.3227. Centre panel, relative change in tumour volume of A375 xenografts over the first 7 days of indicated treatment. Vehicle (n=4), AZD6244 (n=6), AZD8186 (n=6), AZD6244/AZD8186 (n=5); Centre line represents mean. p-values calculated by one-way ANOVA corrected for multiple comparisons (Tukey). Right panel, longitudinal growth of individual tumours from mice harbouring A375 subcutaneous xenografts treated with vehicle (n=5) *vs* AZD6244 (n=6), AZD8186 (n=6), and AZD6244/AZD8186 (n=5). B) Kaplan–Meier overall survival of mice harbouring WM266.4 subcutaneous xenografts treated with vehicle (n=6; median survival, 24 days), cobimetinib/dabrafenib (n=6; median survival, undefined) or cobimetinib/dabrafenib/AZD8186 (n=6; median survival, undefined) or WM266.4 *PREX2*^{ko} D2 subcutaneous xenografts treated with vehicle (n=6; median survival, 25.5 days) or cobimetinib/dabrafenib (n=5; median survival, undefined). p-values calculated by log-rank (Mantel–Cox) test – WM266.4, vehicle vs cobimetinib/dabrafenib, p= 0.000526; vehicle vs cobimetinib/dabrafenib/AZD8186, p=0.000526; cobimetinib/dabrafenib vs cobimetinib/dabrafenib/AZD8186, p=undefined. WM266.4 *PREX2*^{KO} D2, vehicle vs cobimetinib/dabrafenib, p=0.003415. C) Longitudinal growth of individual tumours from mice harbouring WM266.4 subcutaneous xenografts treated with vehicle (n=6), cobimetinib/dabrafenib (n=6) or cobimetinib/dabrafenib/ AZD8186 (n=6) or WM266.4 *PREX2*^{ko} D2 subcutaneous xenografts treated with vehicle (n=6) or cobimetinib/dabrafenib (n=5). D) Relative change in tumour volume of WM266.4 and WM266.4 *PREX2*^{KO} D2 xenografts over the first 7 days of indicated treatment. WM266.4, Vehicle (n=6), cobimetinib/dabrafenib (n=6), cobimetinib/ dabrafenib/AZD8186 (n=6); WM266.4 *PREX2*^{KO} D2, Vehicle (n=6), cobimetinib/dabrafenib (n=5). Centre line represents mean. p-values calculated by one-way ANOVA corrected for multiple comparisons (Tukey). **Continued over.**

Supplementary Fig. S9: Co-targeting of MEK1/2 and p110β has broad therapeutic efficacy in melanoma *in vivo*. (continued) E) Left panel, Kaplan–Meier overall survival of BRAF P53 mice treated with vehicle (n=7; median survival, 14 days), AZD6244 (n=7; median survival, 15 days), AZD8186 (n=5; median survival, 16 days) or AZD6244/AZD8186 (n=8; median survival, 29 days). p-values were calculated by log-rank (Mantel–Cox) test – vehicle vs AZD6244, p=0.0869; vehicle vs AZD8186, p=0.2818; vehicle vs AZD6244/AZD8186, p=0.0002 and AZD6244 vs AZD6244/AZD8186, p=0.2318. Centre panel, relative change in BRAF P53 tumour volume over the first 7 days of indicated treatment. Vehicle (n=5), AZD6244 (n=4), AZD8186 (n=4), AZD6244/AZD8186 (n=7). Centre line represents mean. p-values calculated by one-way ANOVA corrected for multiple comparisons (Tukey). Right panel, longitudinal growth of individual tumours from vehicle-treated (n=5) *vs* AZD6244-treated (n=4), AZD8186-treated (n=4), and AZD6244/AZD8186-treated (n=7) BRAF P53 cohorts. F–I) Pre-treatment tumour volume in WM266.4 xenograft (F), A375 xenograft (G), BRAF PTEN (H), and BRAF P53 (I) cohorts. WM266.4: Vehicle, n=5; AZD6244, n=5; AZD8186, n=5; AZD6244/AZD8186, n=5. A375: Vehicle, n=4; AZD6244, n=6; AZD8186, n=6; AZD6244/AZD8186, n=5. BRAF PTEN: Vehicle, n=8; AZD6244, n=6; AZD8186, n=9; AZD6244/AZD8186, n=11. BRAF P53: Vehicle, n=5; AZD6244, n=4; AZD8186, n=4; AZD6244/AZD8186, n=7. Centre line represents mean. p-values calculated by one-way ANOVA corrected for multiple comparisons (Tukey).

A

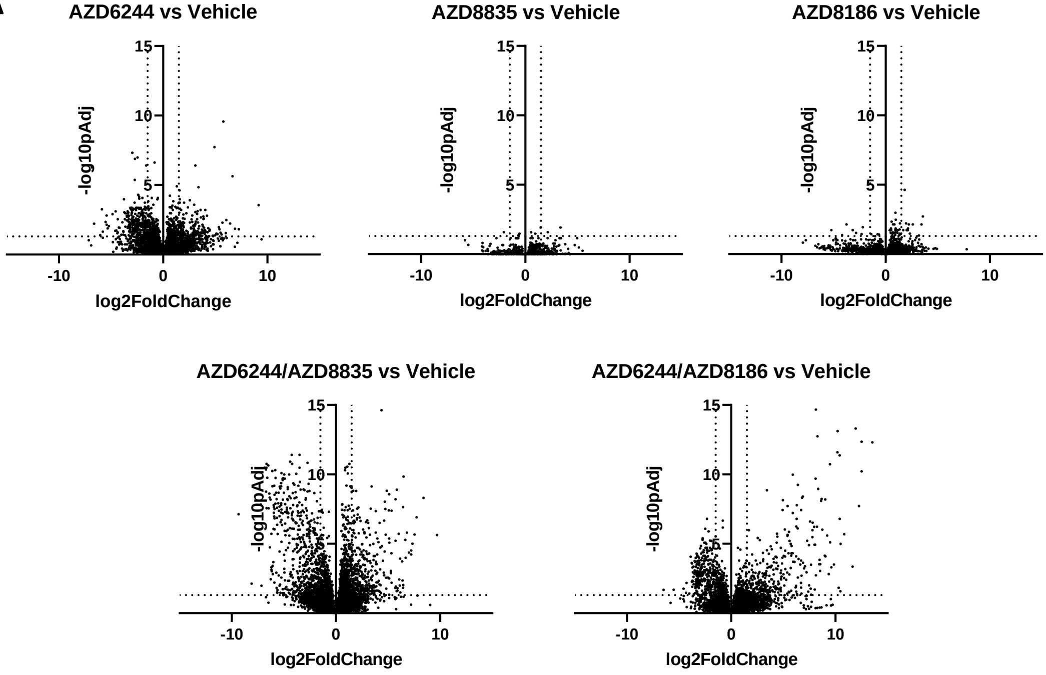

B

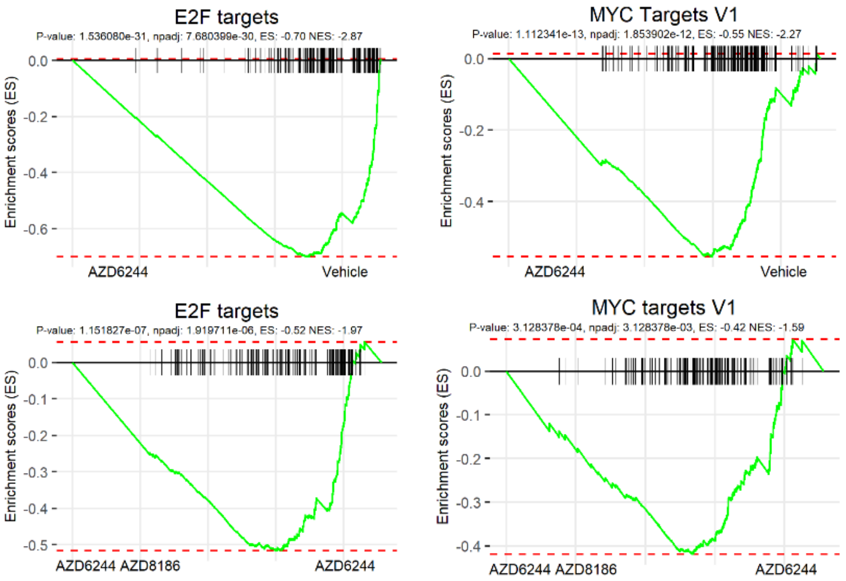

SUPPLEMENTARY 10

Supplementary Fig. S10. Transcriptional profiling identifies key proliferative signatures as targets of combination therapy *in vivo*. A) Volcano plots of log_2FC *vs* $-log_{10}p_{adj}$ of transcripts from BRAF PTEN melanoma *in vivo* following 5-day treatment with AZD6244, AZD8835, AZD8186 and combinations thereof. Horizontal dashed lines represent linear p_{adj}-value of 0.05, vertical dashed lines represent log2FC of 1.5. B) Gene Set Enrichment Analysis (GSEA) plots showing downregulation of cell cycle-associated Hallmark gene sets, including E2F and MYC targets (V1), in xenografted BRAF PTEN tumours treated with AZD6244/AZD8186 combination *vs* AZD6244 monotherapy or AZD6244 monotherapy *vs* vehicle
