# Supplementary material for: Targeting the PREX2/RAC1/PI3Kβ Signaling Axis Confers Sensitivity to Clinically Relevant Therapeutic Approaches in Melanoma
Source: Cancer Res. 2024 Dec 5;85(4):808–24. doi: 10.1158/0008-5472.CAN-23-2814 (PMC11831108; doi:10.1158/0008-5472.CAN-23-2814)
Supplement: Supplementary Table S1 — Reverse Phase Protein Array antibodies [file can-23-2814_supplementary_table_s1_suppst1.docx]

Supplementary Table S1 – Reverse Phase Protein Array antibodies

| **Antibody** | **Supplier** | **Catalogue Number** | **Species** | **Cross-reactivity** |
| --- | --- | --- | --- | --- |
| Histone H2A.X P Ser139 [EP854(2)Y] | Abcam | ab81299 | rabbit | H, M, R |
| Bcl-xl | Abcam/Epitomics | ab32370 | rabbit | H, M, R |
| Bid | Abcam/Epitomics | ab32060 | rabbit | H, (M), (R) |
| Rb | Abcam/Epitomics | ab113074 | rabbit | H, (M), (R) |
| 4E-BP1 P Ser65 (174A9) | Cell Signaling Technology | 9456 | rabbit | H, Mk, (C) |
| 4E-BP1 P Thr37,Thr46 | Cell Signaling Technology | 9459 | rabbit | H, M, R, Mk |
| Akt | Cell Signaling Technology | 9272 | rabbit | H, M, R, Hm, Mk, C, Dm, B, Dg, Pg, GP |
| Akt P Thr308 | Cell Signaling Technology | 2965 | rabbit | H, M, R, Hm, Mk |
| Akt P Ser473 | Cell Signaling Technology | 4060 | rabbit | H, M, R, Hm, Mk, Dm, Z, B |
| ATM/ATR Substrate P Ser/Thr | Cell Signaling Technology | 2851 | rabbit | H, M, R, Mk, All |
| Bad P Ser112 | Cell Signaling Technology | 9291 | rabbit | H, M, R, Mk |
| Bad P Ser136 | Cell Signaling Technology | 9295 | rabbit | (H), M, (R) |
| Bim P Ser69 | Cell Signaling Technology | 4585 | rabbit | H, M, (R), (Mk), (Dg) |
| BRCA1 | Cell Signaling Technology | 9010 | rabbit | H |
| Caspase 3 | Cell Signaling Technology | 9662 | rabbit | H, M, R, Mk |
| Caspase 3 cleaved | Cell Signaling Technology | 9664 | rabbit | H, M, R, Mk, (B), (Dg), (Pg) |
| cdc25c P Ser216 | Cell Signaling Technology | 4901 | rabbit | H, Mk |
| CDK1 (cdc2) | Cell Signaling Technology | 9112 | rabbit | H, M, R |
| Chk2 P Thr68 | Cell Signaling Technology | 2661 | rabbit | H, Mk |
| c-Jun P Ser73 | Cell Signaling Technology | 9164 | rabbit | H, M, R, Mk |
| c-Myc | Cell Signaling Technology | 5605 | rabbit | H, M, R, (Dg), (Pg) |
| Cyclin D1 | Cell Signaling Technology | 2922 | mouseIgG2a | H, M, R |
| Cyclin D1 P Thr286 | Cell Signaling Technology | 3300 | rabbit | H, (Mk) |
| EGFR P Tyr1173 | Cell Signaling Technology | 4407 | rabbit | H, M, R |
| ErbB-1/EGFR | Cell Signaling Technology | 2232 | rabbit | H, M, R, Mk |
| ErbB-3/Her3/EGFR | Cell Signaling Technology | 4754 | rabbit | H, (M), (R) |
| IGF-1R beta | Cell Signaling Technology | 3027 | rabbit | H, M, R, Mk |
| IRS-1 | Cell Signaling Technology | 2382 | rabbit | H, M, R |
| MAPKAPK-2 P Thr334 | Cell Signaling Technology | 3041 | rabbit | H, M, R, Mk |
| MEK1/2 | Cell Signaling Technology | 9122 | rabbit | H, M, R, Mk, (C), Dm, (X) |
| MEK1/2 P Ser217/221 | Cell Signaling Technology | 9154 | rabbit | H, M, R, Mk, (C), Dm |
| mTOR (7C10) | Cell Signaling Technology | 2983 | rabbit | H, M, R, Mk, (Hr) |
| mTOR P Ser2448 | Cell Signaling Technology | 2971 | rabbit | H, M, R, Mk |
| p21 CIP/WAF1 | Cell Signaling Technology | 2946 | mouseIgG2a | H, Mk |
| p38 MAPK | Cell Signaling Technology | 9212 | rabbit | H, M, R, Mk, (C), GP |
| p38 MAPK PThr180,Tyr182 | Cell Signaling Technology | 9211 | rabbit | H, M, R, (Hm), Mk, Dm, (Z), (B), Pg, Sc |
| p44/42 MAPK (ERK1/2) | Cell Signaling Technology | 9102 | rabbit | H, M, R, Hm, Mk, Mi, Z, B, Pg, Sc |
| p44/42 MAPK (ERK1/2) P Thr202/Thr185,Tyr204/Tyr187 | Cell Signaling Technology | 4370 | rabbit | H, M, R, Hm, Mk, (C), Mi, Dm, Z, B, Dg, Pg, Sc, (Ce) |
| p53 | Cell Signaling Technology | 9282 | rabbit | H, Mk |
| p53 P Ser15 | Cell Signaling Technology | 9284 | rabbit | H, M, R, Mk, (Mi), (B), (Pg) |
| p70 S6 Kinase P Thr389 | Cell Signaling Technology | 9205S | rabbit | H, M, R, Mk |
| p70 S6 Kinase P Thr421,Ser424 | Cell Signaling Technology | 9204 | rabbit | H, M, R, Mk |
| p90 S6 kinase (Rsk1-3) P Thr359,Ser363 | Cell Signaling Technology | 9344 | rabbit | H, M, R, Mk |
| PARP | Cell Signaling Technology | 9542 | rabbit | H, M, R, Mk |
| PARP cleaved Asp214 | Cell Signaling Technology | 9541 | rabbit | H |
| PDK-1 | Cell Signaling Technology | 3062 | rabbit | H, M, R, Mk, (C) |
| PDK-1 P Ser241 | Cell Signaling Technology | 3061 | rabbit | H, M, R |
| PI3 Kinase p110-alpha | Cell Signaling Technology | 4249 | rabbit | H, M, R, B |
| PLC-gamma1 | Cell Signaling Technology | 2822 | rabbit | H, M, R |
| PLC-gamma1 P Tyr783 | Cell Signaling Technology | 2821 | rabbit | H, M, R |
| PTEN | Cell Signaling Technology | 9552 | rabbit | H, M, R, Hm, Mk, (C) |
| PTEN P Ser380,Thr382,Thr383 | Cell Signaling Technology | 9554 | rabbit | H, M, R |
| Puma | Cell Signaling Technology | 4976 | rabbit | H, (Mk) |
| Raf P Ser259 | Cell Signaling Technology | 9421 | rabbit | H, M, R, Mk, (C), X |
| Raf P Ser338 | Cell Signaling Technology | 9427 | rabbit | H, M, R, Mk |
| Rb P Ser807,Ser811 | Cell Signaling Technology | 9308 | rabbit | H, (M), R, Mk |
| Rb P Ser780 | Cell Signaling Technology | 9307 | rabbit | H, R, Mk |
| S6 Ribosomal Protein | Cell Signaling Technology | 2217 | rabbit | H, M, R, Mk, (Pg) |
| S6 Ribosomal protein P Ser235,Ser236 | Cell Signaling Technology | 2211 | rabbit | H, M, R, Mk, (C), (X), Sc |
| S6 Ribosomal protein p Ser240,Ser244 | Cell Signaling Technology | 2215 | rabbit | H, M, R, Mk, (C), (X), Z |
| SHP2 P Tyr542 | Cell Signaling Technology | 3751 | rabbit | H, M, R, (Mk), (C), (X) |
| Survivin | Cell Signaling Technology | 2808 | rabbit | H, M, R |
| Tsc-2 (Tuberin) | Cell Signaling Technology | 3612 | rabbit | H, M, R, Mk |
| Tuberin P S1387 | Cell Signaling Technology | 5584 | rabbit | H, M, R, Mk |
| VEGFR P Tyr951 | Cell Signaling Technology | 4991 | rabbit | H, M |
| Bak | Epitomics | 1542-1 | rabbit | H (M), (R) |
| Bax | Epitomics | 1063 | rabbit | H, M, R, |
| Bcl-2 | Epitomics | 1017-1 | rabbit | H |
| Bim | Epitomics | 1036 | rabbit | H, M, R |
| c-Jun N-term | Epitomics | 1254-1 | rabbit | H, M, R |
| c-Myc P Thr58,Ser62 | Epitomics | 1203-1 | rabbit | H, R |
| MAPKAPK-2 | Epitomics | 1497-1 | rabbit | H, M, R |
| EGFR P Tyr1086 | Invitrogen | 369700 | rabbit | H, M, R |
| FRA1 (R20) | Santa Cruz | sc-605 | rabbit | H, M, R |
| p21 CIP/WAF1 p Thr145 | Santa Cruz | sc-20220-R | rabbit | H, M, R, bd, Pg, Dg, |
| Met P Tyr1234 | Signalway | 11227-1 | rabbit | H, M, R |
| Met P Tyr1349 | Signalway | 11238 | rabbit | H, M, R |
