# Supplementary material for: Targeting the PREX2/RAC1/PI3Kβ Signaling Axis Confers Sensitivity to Clinically Relevant Therapeutic Approaches in Melanoma
Source: Cancer Res. 2024 Dec 5;85(4):808–24. doi: 10.1158/0008-5472.CAN-23-2814 (PMC11831108; doi:10.1158/0008-5472.CAN-23-2814)
Supplement: Supplementary Table S2 — Cancer subtypes abbreviations [file can-23-2814_supplementary_table_s2_suppst2.docx]

| **TCGA PanCancer Atlas** | | **MSK IMPACT** | |
| --- | --- | --- | --- |
| SKCM | Skin Cutaneous Melanoma | SKCM | Melanoma |
| STAD | Stomach Adenocarcinoma | BRCA | Breast Cancer |
| UCS | Uterine Carcinosarcoma | BLCA | Bladder Cancer |
| LIHC | Liver Hepatocellular Carcinoma | HNSC | Head and Neck Cancer |
| LUSC | Lung Squamous Cell Carcinoma | HPB | Hepatobiliary Cancer |
| UCEC | Uterine Corpus Endometrial Carcinoma | CRC | Colorectal Cancer |
| EAC | Esophageal Carcinoma | OGCA | Esophagogastric Cancer |
| COAD | Colorectal Adenocarcinoma | NSCLC | Non-Small Cell Lung Cancer |
| LUAD | Lung Adenocarcinoma | IPMN | Intraductal Papillary Mucinous Neoplasm |
| BLCA | Bladder Urothelial Carcinoma | UEC | Uterine Endometrioid Carcinoma |
| BRCA | Breast Invasive Carcinoma | OV | Ovarian Cancer |
| PRAD | Prostate Adenocarcinoma | EC | Endometrial Cancer |
| OV | Ovarian Serous Cystadenocarcinoma | BC | Bone Cancer |
| HNSC | Head and Neck Squamous Cell Carcinoma | STS | Soft Tissue Sarcoma |
| PAAD | Pancreatic Adenocarcinoma | LCA | Lung Cancer |
| SARC | Sarcoma | PaCA | Pancreatic Cancer |
| DLBC | Diffuse Large B-Cell Lymphoma | PrCA | Prostate Cancer |
| CESC | Cervical Squamous Cell Carcinoma | CCA | Cervical Cancer |
| CHOL | Cholangiocarcinoma | MBL | Mature B-Cell Lymphoma |
| ACC | Adrenocortical Carcinoma | RCC | Renal Cell Carcinoma |
| GBM | Glioblastoma Multiforme | EmCA | Embryonal Tumour |
| PCPG | Pheochromocytoma and Paraganglioma | MBCN | Mature B-Cell Neoplasms |
| AML | Acute Myeloid Leukaemia | GLI | Glioma |
| TGCT | Testicular Germ Cell Tumours |  |  |
| UVM | Uveal Melanoma |  |  |
| KIRC | Kidney Renal Clear Cell Carcinoma |  |  |
| LGG | Brain Low Grade Glioma |  |  |
| KIRP | Kidney Renal Papillary Cell Carcinoma |  |  |
| THCA | Thyroid Carcinoma |  |  |

Supplementary Table S2 – Cancer Subtype Abbreviations
